# Midgut serine proteinases participate in dietary adaptations of the castor (Eri) silkworm *Samia ricini* Anderson transferred from *Ricinus communis* to an ancestral host, *Ailanthus excelsa* Roxb

**DOI:** 10.3389/finsc.2023.1169596

**Published:** 2023-08-10

**Authors:** Sochanngam Kashung, Parul Bhardwaj, Mahaswetta Saikia, Sudeshna Mazumdar-Leighton

**Affiliations:** Plant-Biotic Interactions Lab, Department of Botany, University of Delhi, Delhi, India

**Keywords:** host plant choice, non-mulberry silkworm, nutrition, digestive physiology, serine proteinases, larval gut gene expression, performance, domestication

## Abstract

Dietary change influenced the life-history traits, nutritional utilization, and midgut serine proteinases in the larvae of the domesticated polyphagous *S. ricini*, transferred from *R. communis* (common name: castor; family Euphorbiaceae; the host plant implicated in its domestication) to *A. excelsa* (common name: Indian tree of heaven; family Simaroubaceae; an ancestral host of wild *Samia* species). Significantly higher values for fecundity and body weight were observed in larvae feeding on *R. communis* (Scr diet), and they took less time to reach pupation than insects feeding on *A. excelsa* (Scai diet). Nevertheless, the nutritional index for efficiency of conversion of digested matter (ECD) was similar for larvae feeding on the two plant species, suggesting the physiological adaptation of *S. ricini* (especially older instars) to an *A. excelsa* diet. *In vitro* protease assays and gelatinolytic zymograms using diagnostic substrates and protease inhibitors revealed significantly elevated levels (*p* ≤ 0.05) of digestive trypsins, which may be associated with the metabolic costs influencing slow growth in larvae feeding on *A. excelsa*. RT-PCR with semidegenerate serine proteinase gene-specific primers, and cloning and sequencing of 3′ cDNA ends identified a large gene family comprising at least two groups of putative chymotrypsins (i.e., Sr I and Sr II) resembling invertebrate brachyurins/collagenases with wide substrate specificities, and five groups of putative trypsins (i.e., Sr III, Sr IV, Sr V, Sr VII, and Sr VIII). Quantitative RT-PCR indicated that transcripts belonging to the Sr I, Sr III, Sr IV, and Sr V groups, especially the Sr IV group (resembling achelase I from *Lonomia achelous*), were expressed differentially in the midguts of fourth instars reared on the two plant species. Sequence similarity indicated shared lineages with lepidopteran orthologs associated with expression in the gut, protein digestion, and phytophagy. The results obtained are discussed in the context of larval serine proteinases in dietary adaptations, domestication, and exploration of new host plant species for commercial rearing of *S. ricini*.

## Introduction

*Samia ricini* (syn. *Samia cynthia ricini* Drury, *Phalaena ricini* Jones) is a multivoltine economically useful species of Lepidoptera, which was domesticated in the Indo-Burmah/Myanmar region for its cocoon silk fibers and edible pupae (1, 2). The larvae of *S. ricini* are generalists *par excellence* and can feed on more than 30 species of plants belonging to disparate taxonomic families, such as Euphorbiaceae, Simaroubaceae, Araliaceae, Caricaceae, Brassicaceae, and Rutaceae (1, 3). Despite it being a generalist feeder, the larvae of *S. ricini* have been reared almost exclusively on *Ricinus communis* for centuries (1, 4). Not much information is available on the history behind the anthropogenic choice of *R. communis* as a larval host plant for the domestication of *S. ricini*. The larvae of *S. ricini* can also feed on *Ailanthus excelsa* Roxb., a perennial tree species with copious foliage (1, 5). Several species of the genus *Ailanthus* are primary host plants for the larvae of saturniids such as *Samia cynthia cynthia*, the ailanthus silkmoth found in Southeast Asia (3, 6). In fact, *A. excelsa* is a natural host plant for *Samia canningi* (Hutton), an extant wild species from north-east India, which is reported to be a progenitor of *S. ricini* (7). There are probably multiple mechanisms that influence the adaptations of *S. ricini* larvae to herbivory and utilization of diverse host plants (8, 9).

Protein digestion in the Lepidoptera is a complex process carried out by hydrolases in the alkaline larval gut. Among insect digestive enzymes, serine proteases (EC 3.4.21) have been well characterized (10). Serine proteinases contain a catalytic triad comprising H57, D102, and S195 residues (numbering after bovine chymotrypsinogen) embedded within semiconserved sequence motifs (11, 12). In addition to digestion, serine proteinases participate in several biological processes, such as fertilization, embryonic development, blood coagulation, homeostasis, and immunity in *Animalia* (11–13). It is probable that digestive serine proteinases in phytophagous Lepidoptera have evolved in response to selection pressures imposed by substrates encountered in ingested host plant tissues over space and time (14, 15). Insect gut proteases interact with dietary plant protease inhibitors (PPIs) that are induced as “direct” defense responses of plants to herbivory (16). Adaptive responses of lepidopteran larvae to dietary PPIs are complex and can involve the hyperproduction of some midgut serine proteinases, rapid transcription of PPI-insensitive serine proteinases in the midgut and/or proteolysis of the PPI (17), thus contributing to the utilization of one or most host plants. In contrast to the better-studied digestive serine proteases of the domesticated but monophagous “mulberry” silkworm, *Bombyx mori* L., the physiology and molecular characterization of midgut proteases in the domesticated, polyphagous *S. ricini* silkworm have not been examined in detail.

Diverse groups of serine proteases are expressed in the guts of silkworms such as *B. mori*, *Antheraea pernyi*, and *Antheraea assamensis* (18–21). The digestive serine proteases in *S. ricini* larvae are mostly uncharacterized. The large datasets generated from gut transcriptomes of Lepidoptera from different feeding guilds/taxonomic families indicate that the transcripts encoding serine proteinases typically belong to large families, some of which show differential expression in response to dietary changes during larval development (22–25). The profile changes of larval gut serine proteinases in response to ingested PPI can occur at the same time as adaptations to secondary metabolites characteristic of the host plant family (23, 26). The changes in profile of larval gut serine proteases often accompanies a rapid response of lepidopteran larvae to nutritive stress—for example, the ingestion of toxic proteins not normally encountered in native host plants, such as the endotoxins of *Bacillus thuringiensis* (Bt) (27–29)—and on infection by various pathogens (18, 20, 21, 30). The quantum of particular differentially expressed gut serine proteases may or may not lead to the efficient digestion of dietary constituents. Fitness costs, such as delayed larval growth and/or low weight gain can be incurred in insects with poor metabolic adaptations to a given host plant type (31–34).

In this study, life-history traits, nutritional indices, and digestive serine proteases were evaluated for various instars of *S. ricini* larvae feeding on a diet of *R. communis* and transferred to an *A. excelsa* diet. Transcripts encoding putative serine protease cDNAs were identified and quantified in the midgut tissues of fourth instars feeding on the two host types. The sequence homology of the *S. ricini* proteases with closely related orthologs belonging to distinct groups/lineages was also examined. Such studies on digestive serine proteinases and their genes provide an interesting platform for future research on economically beneficial silkworms that provide fiber, food, feed, and alternative livelihood to indigenous communities. An understanding of how larval gut proteinases in the polyphagous *S. ricini*, domesticated over centuries on *R. communis*, respond to a dietary shift onto an ancestral host, such as *A. excelsa*, has practical implications for exploring alternative hosts for the commercial rearing of *S. ricini.*

## Materials and methods

### Measurement of life history traits of *S. ricini* reared on diets of *R. communis* and *A. excelsa*

For this study, disease-free egg layings of *S. ricini* (yellow plain “eco-race”) were obtained from the Department of Sericulture, Government of Manipur, India. First instars from egg clusters of females reared on *R. communis* were carefully transferred to either *R. communis* (Scr) or *A. excelsa* (Scai) diets. Larvae were reared at an ambient temperature of 25 ± 2°C, a relative humidity of 80% ± 5%, and a photoperiod of 14: 10 hours light: dark at the Insect Rearing Facility, Department of Botany, University of Delhi, Delhi. The provenances of insects used in this study are available as GenBank accessions #KX951450 and #EU126821 for *EF1*-*α* gene and actin gene, respectively. Host plants *R. communis* and *A. excelsa* were sampled from the Department of Botany Garden, University of Delhi (28°40′25.48′′N, 77°12′34.97′′E and 28°41′04.36′′N, 77°12′27.04′′E, respectively). The fully expanded mature leaves of *R. communis* (20.2 ± 1.61 mg/mL total protein per gram of leaf tissues) were used to feed the third, fourth, and fifth instars. In the case of *A. excelsa*, mature leaves (18.3 ± 0.33 mg/mL total protein per gram of leaf tissues) from a three-year old tree were collected and used as food for the larvae. The provenances of the plant materials used in this study are available as #KX951434 and #KX951435 for the *rbcL* gene from *R. communis* and *A. excelsa*, respectively.

–Life-history traits that were studied included the duration of each larval instar (measured at each new molt); larval weight at the beginning of each instar (measured as fresh weight in grams); head size of larva per instar (measured immediately after each molt in mm using a Vernier caliper); pupal weight; cocoon shell weight (measured in grams at the end of spinning cycle); number of eclosing moths per egg cluster; and the weight of male and female moths per egg cluster. For the estimation of realized fecundity, the average number of eggs oviposited per female was determined using three non-sib moths. No effort was made to determine the number of eggs retained by each female moth (unrealized fecundity). This number is reported as an average of 3%–10% of the potential (realized + unrealized) fecundity in females from commercial grainages in India (35). The egg hatchability (number of neonates emerging per egg cluster) and survival rate (number of larvae that completed the life cycle per number of neonates brushed) were determined using the methods described by Shifa et al. (36). At least 50 larvae selected randomly from three independent non-sib egg clusters were used as biological replicates for experiments on life-history traits, except for the measurements for realized fecundity, for which three unrelated female moths reared were used. The data were analyzed statistically using the Mann–Whitney test for two independent samples, and one-way ANOVA by least significant difference (LSD) and Tukey’s honestly significant difference (HSD) for more than two independent samples using IBM spss Statistics version 21 (IBM Corporation, Armonk, NY, USA).

### Nutritional indices of *S. ricini* larvae from feeding assays and continuous rearing on *R. communis* and *A. excelsa*

The host plant utilization efficiencies were determined in accordance with the methods described by Waldbauer (37) and Scriber and Slansky (38). Feeding assays were carried out in the form of “no-choice, fixed-time” experiments for 12 hours to measure larval weight gained, weights of food consumed and food left uneaten, and the weight of fecal matter for each instar (39). The stadia of larval development were measured by size of the head capsule immediately after each molt, as described above. In a control experiment, nutritional indices were also determined for larvae reared from neonate up to the cocoon spinning stage on only *R. communis* (Scr) and *A. excelsa* (Scai) diets. At least three biological replicates were used, and each setup contained 10 larvae (*n* = 10*3 = 30 larvae). The relative growth rate (RGR), efficiency of conversion of ingested food (ECI), approximate digestibility (AD), and efficiency of conversion of digested food (ECD) were calculated on a dry weight basis. Correction factors (40) for the water loss from fresh weights of leaves, larvae, and frass were calculated for the measurement of nutritional indices using the method described by Kumar et al. (39). Formulae used for determination of nutritional indices were as follows:


(1) $$\begin{array}{l} \text{Efficiency of conversion of ingested food (ECI)}=\text{(weight gained}*100/\\ \text{weight of food ingested} \end{array}$$

(2) $$\begin{array}{l} \text{Approximate digestibility (AD)}=\text{(weight of food ingested}-\text{weight of feces)}*100/\\ \text{weight of food ingested} \end{array}$$

(3) $$\begin{array}{l} \text{Efficiency of conversion of digested food (ECD)}=\text{weight gained}*100/\\ \text{(weight of food ingested}-\text{weight of feces)} \end{array}$$

(4) $$\begin{array}{l} \text{Relative growth rate (RGR)}=\text{weight gained}/\text{duration of feeding period}*\\ \text{mean weight during feeding period} \end{array}$$

The data were arcsine transformed before statistical analysis by one-way ANOVA was carried out as described above.

### Identification of midgut proteinases using *in vitro* spectrophotometric assays with diagnostic substrates, protease inhibitors, and gelatin zymography

Midgut tissues (including the lumen) were carefully dissected from actively feeding third, fourth, and fifth instars before homogenization in cold 100 mM HEPES (*N*-2- hydroxyethylpiperazine-*N*′-2-ethanesulfonic acid) buffer at pH 8.0. *In vitro* assays for trypsin and chymotrypsin activities were conducted using the method described by Broadway (31), using amidolytic substrates *N*α-benzoyl L-arginine 4-nitroanilide hydrochloride (B-A-pNA; catalogue# B4875) and *N*-succinyl-alanine-alanine-proline-phenylalanine-*p*-nitroanilide (Suc-AAPF-pNA; catalogue# S7388), respectively. Two heterologous inhibitors of plant origin, that is, soybean trypsin inhibitor or STI (1mg/mL, catalogue# T9128), and soybean Bowman–Birk inhibitor or SBBI, a dual trypsin–chymotrypsin inhibitor (1 mg/mL; catalogue# T977) were tested. The assays were conducted with glycine–sodium hydroxide (NaOH) buffer (100 mM, pH 10.0) with or without inhibitors. All reagents were purchased from Sigma-Aldrich Co., Missouri, USA, unless mentioned otherwise. The total protein estimations were determined using the Bio-Rad Protein Assay (catalogue# 500–0006; Bio-Rad Laboratories, Inc., CA, USA). All experiments were conducted in triplicate with at least three biological replicates.

Midgut extracts were subjected to 10% gelatin zymography using the methods described by Michaud (41) and Oppert et al. (42). The samples were also preincubated with STI (2 mg/mL) and SBBI (2 mg/mL) for 15 minutes at 37°C before substrate zymography was carried out. In addition, gelatinolytic zymograms were prepared by preincubating gut extracts of fourth instars feeding continuously on *R. communis* with a battery of protease inhibitors: STI (2 mg/mL); SBBI (2 mg/mL); *N*α-tosyl-L-lysine chloromethyl ketone hydrochloride or TLCK, a synthetic diagnostic inhibitor of mammalian trypsins, (9 mM; catalogue #T7254); E-64, a cysteine protease inhibitor (1 mg/mL; catalogue #E3132); *N*-acetyl-L-leucyl-L-leucyl-L-argininal hemisulfate salt or leupeptin, a microbial tripeptide inhibitor of mammalian trypsins and cysteine protease (1 mg/mL; catalogue #L9783); aprotinin, a peptidyl inhibitor of mammalian trypsins, (1 mg/mL; catalogue #A1153); ethylenediaminetetraacetic acid or EDTA, a metalloprotease inhibitor (3 mM); and pepstatin, a microbial aspartic protease inhibitor (1 mg/mL; catalogue #P4265).

### Identification and quantification of midgut serine protease genes expressed in fourth-instar *S. ricini* feeding on *R. communis* and *A. excelsa*

Total RNA was isolated from midgut tissues of fourth instars feeding on the two host plant types (43), and cDNAs were synthesized using a GeneAmp^®^ RNA PCR core kit (Part No. N808–0143; Applied Biosystems Inc., CA, USA) in accordance with the vendor’s instructions using a DNA Engine^®^ Peltier Thermal Cycler (Model# PTC0200, Bio-Rad Laboratories, Inc.). Semidegenerate serine protease-specific oligonucleotide primers (**Table 1**) were used for RT-PCR and 3′ rapid amplification of 3′ cDNA ends (RACE) with anchored oligo dT_18_ primers using the method described by Mazumdar-Leighton et al. (44). All amplicons were cloned into the pGEM^®^-T Easy Vector system (catalogue# A1360; Promega Corporation, Madison, WI, USA) in accordance with the vendor’s instructions. Unique open reading frames (ORFs) were identified from the restriction digestion patterns of the clones using the method described by Mazumdar-Leighton and Broadway (45). At least three clones representing each unique ORF were sequenced on both strands (Macrogen, Inc., Seoul, Democratic People’s Republic of Korea). The sequences (including and excluding the primers) were used as queries in BLAST homology searches (46). Putative serine protease sequences were aligned with Clustal W available in MEGA 7.0 (www.megasoftware.net/). Gene-specific primers for distinct groups of transcripts were designed (**Table 1**) and their application was confirmed by RT-PCR amplification, cloning, and sequencing of cloned amplicons before performing qRT-PCR was carried out. The qRT-PCR experiments were conducted using the methods described by Livak and Schmittgen (47) and Saikia et al. (19). Briefly, iQ SYBR^®^ Green Supermix (catalog # 1708882; Bio-Rad Laboratories, Inc.) was used with gene-specific primers (150 ng/µl; **Table 1**) and equal amounts of the mRNA template (100 ng/µL). The qRT-PCR experiments were conducted using a CFX Connect Real-Time PCR Detection System (Bio-Rad Laboratories, Inc.). The profiles comprised an initial denaturation at 95°C for 1 minute followed by 40 cycles of denaturation at 95°C for 30 seconds, annealing at 55°C for 30 seconds, and extension at 72°C for 30 seconds. All qRT-PCR experiments used RNA obtained from at least three biological replicates of gut tissues from larvae originating from different egg clusters and mated parents. The templates were quantified on a Multiskan™ GO Microplate Spectrophotometer (catalog# 51119200; Thermo Fisher Scientific, Waltham MA, USA). The relative gene expression levels were determined with reference to a household gene encoding the *S. ricini* elongation factor 1 alpha, SrEF1-α (GenBank accession KX951450) in larvae feeding on the *A. excelsa* (Scai) diet. Data from various experiments were analyzed statistically using one-way ANOVA and a HSD test using spss Statistics version 21 (IBM Corporation). **Table S1** provide a list if sequences from this study that were submitted to the National Center for Biotechnology Information (NCBI) GenBank (www.ncbi.nlm.nih.gov).

**Table 1:** List of primers used in the study.

Primers (amino acid)	Sequence information
DmTF (IVTAAH)	5′ TCGAATTCATTGTGACCGCCGCTCAYTG 3′
DmTR (KDACQGDS)	5′ TCTCTAGAGTCACCCTGGCAGGCRTCYTT 3′
SerPR (GDSGGP)	5′ TATCTAGATGGGCCACCGGAATCCCCCTG 3′
RcTR (RDACQGDS)	5′ TCTCTAGAGTCACCCTGGCAGGCGTCCCG 3′
Oligo dT_18_MA	5′ TTTTTTTTTTTTTTTTTT(A/C/G)A 3′
Oligo dT_18_MC	5′ TTTTTTTTTTTTTTTTTT(A/C/G)C 3′
Oligo dT_18_MG	5′ TTTTTTTTTTTTTTTTTT(A/C/G)G 3′
Oligo dT_18_MT	5′ TTTTTTTTTTTTTTTTTT(A/C/G)T 3′
Sr1F	5′ AACGACATCGCTATGCTT 3′
Sr1R	5′ ATCTAAAACTAAGTTACC 3′
Sr2F	5′ AATGACATCTCCCTGTTG 3′
Sr2R	5′ TGTTGTGCCGAAGTTAAG 3′
Sr3F	5′ AACGATGTAGCGGTCGTT 3′
Sr3R	5′ GCCGGGTTGAGTCTTCAG 3′
Sr4F	5′ AACGACATCGCTGTCATG 3′
Sr4R	5′ GGCGGTGATAGTGTCACC 3′
Sr5F	5′ AATGACGTCGCTATCTTA 3′
Sr5R	5′ AGCAGTGACAAAGCGTAT 3′
Sr7F	5′ GGCGACGTCAGCGTTATC 3′
Sr7R	5′ GCCCGGTGTAGGTAGAGT 3′
*EF1-α F*	5′ ACATTGTCGTCATTGGACAC 3′
*EF1-α R*	5′ AGTGTGAAAGCGAGAAGAGC 3′

Where Y = C + T; R = A + G; N = A + T + C + G; M = A + C + G.

### Sequence relatedness of *S. ricini* serine proteinases with closely related lepidopteran orthologs

Sequence relatedness of putative serine proteinases from *S. ricini* was explored using a BLAST-p homology search (46) and the NCBI non-redundant (nr) database (release 245). Lepidopteran orthologs, especially those with functional annotations and reports associated with dietary changes, and close relatives from the Saturniidae family, were identified using a cutoff value of at least 65% identity and 96% query coverage at the amino acid level. Only one representative isoform from any group of homologs (sharing > 95% sequence identity) was usually retained (**Table S1**). A Bayesian tree was constructed using BEAST v1.10.4 software (https://beast-dev.github.io/beast-mcmc/). Parameters included: substitution model—Whelan and Goldman (WAG) (www.ebi.ac.uk/goldman-srv/WAG/); site heterogeneity model—gamma + invariant sites, with the number of gamma categories as four; tree prior—coalescent: constant; and Markov chain Monte Carlo (MCMC)—length of change: 100,000,000. The tree was decorated using FigTree v1.4.3 (http://tree.bio.ed.ac.uk/software/figtree/). Multiple sequence alignments of serine proteases from *S. ricini*, selected lepidopteran orthologs, and invertebrate/mammalian homologs with known/resolved protein structures from the Protein Data Bank (PDB) (www.ebi.ac.uk/merops) were obtained using Clustal Omega (www.ebi.ac.uk/Tools/msa/clustalo/). The consensus of the aligned sequences was depicted using Jalview (www.jalview.org/).

## Results

### *S. ricini* larvae feeding on *R. communis* show superior performance for most life-history traits but larvae feeding on *A. excelsa* also complete life cycle and spin cocoons

**Figure 1:**
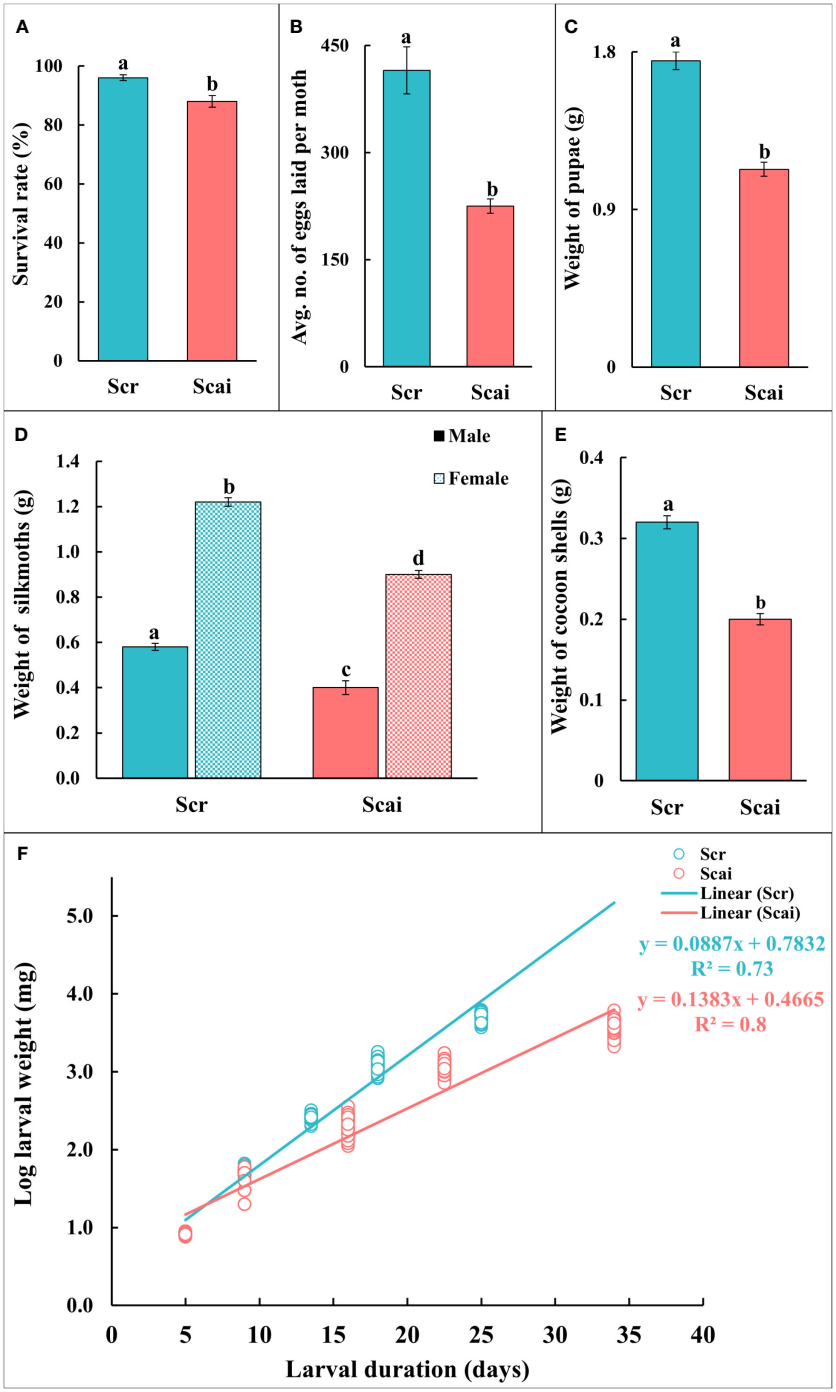
Life history traits of *Samia ricini* reared on *Ricinus communis* (Scr) and *Ailanthus excelsa* (Scai) diets. **(A)** Survival rate = percentage of insects reaching adult stage; **(B)** realized fecundity = average number of eggs laid by a moth; **(C)** fresh weight (g) of pupae; **(D)** fresh weight (g) of newly eclosed silkmoths; and **(E)** cocoon shell weight (g). The sample size (*n*) for each experiment on life history was ≥50; except for realized fecundity where three non-sib females from each diet were used. Bars (Scr, blue; Scai, red) depict mean ± SE. Significant differences at a *p*-value ≤0.05 are denoted by different letters; and **(F)** regression plot shows the log-larval fresh weight (mg) in relation to larval duration (days) of *S. ricini* fed on *R. communis* (Scr, blue line) and *A. excelsa* (Scai, red line) diets. The weight of individual larva fed on a Scr diet (blue) and Scai diet (red) is shown as a circle. The relation was linearized using the log-larval weight. *R* ^2^-values are provided.

**Figure 1A** shows that the percentage survival of insects reared on both diets was greater than 90%. However, a significantly larger number of insects (*p* ≤ 0.05) reared on *R. communis* (Scr) reached maturity than those reared on *A. excelsa* (Scai). The realized fecundity (**Figure 1B**) and weight of pupae (**Figure 1C**) were significantly higher (*p* ≤ 0.05) for insects reared on *R. communis* (Scr) than for those reared on a diet of *A. excelsa* (Scai). Both female and male insects reared on *R. communis* were heavier (significant at *p* ≤ 0.05) than those reared on *A. excelsa* (**Figure 1D**). The weights of cocoon shells (on insect emergence), an economically important parameter, were significantly higher for insects reared on Scr than those reared on Scai (**Figure 1E**). The traits for which no significant differences were observed between insects feeding on the Scr and Scai diets included the percentage eclosion of moths emerging from cocoons and the percentage of neonates hatching from eggs. A straight line was observed from a regression analysis of log-larval weight gained on the two plant diets and time taken for larval development (**Figure 1F**; *R* ^2^ = 0.734 for larvae fed the Scr diet and *R* ^2^ = 0.82 for those fed the Scai diet). Different slopes of the two regression lines indicated that insects fed the *A. excelsa* diet took longer to complete larval development and gained less weight than those fed the *R. communis* diet.

### Larval age and type of host plant differently influence nutritional indices for *S. ricini* feeding on *R. communis* and *A. excelsa* in no-choice fixed-time assays

**Figure 2:**
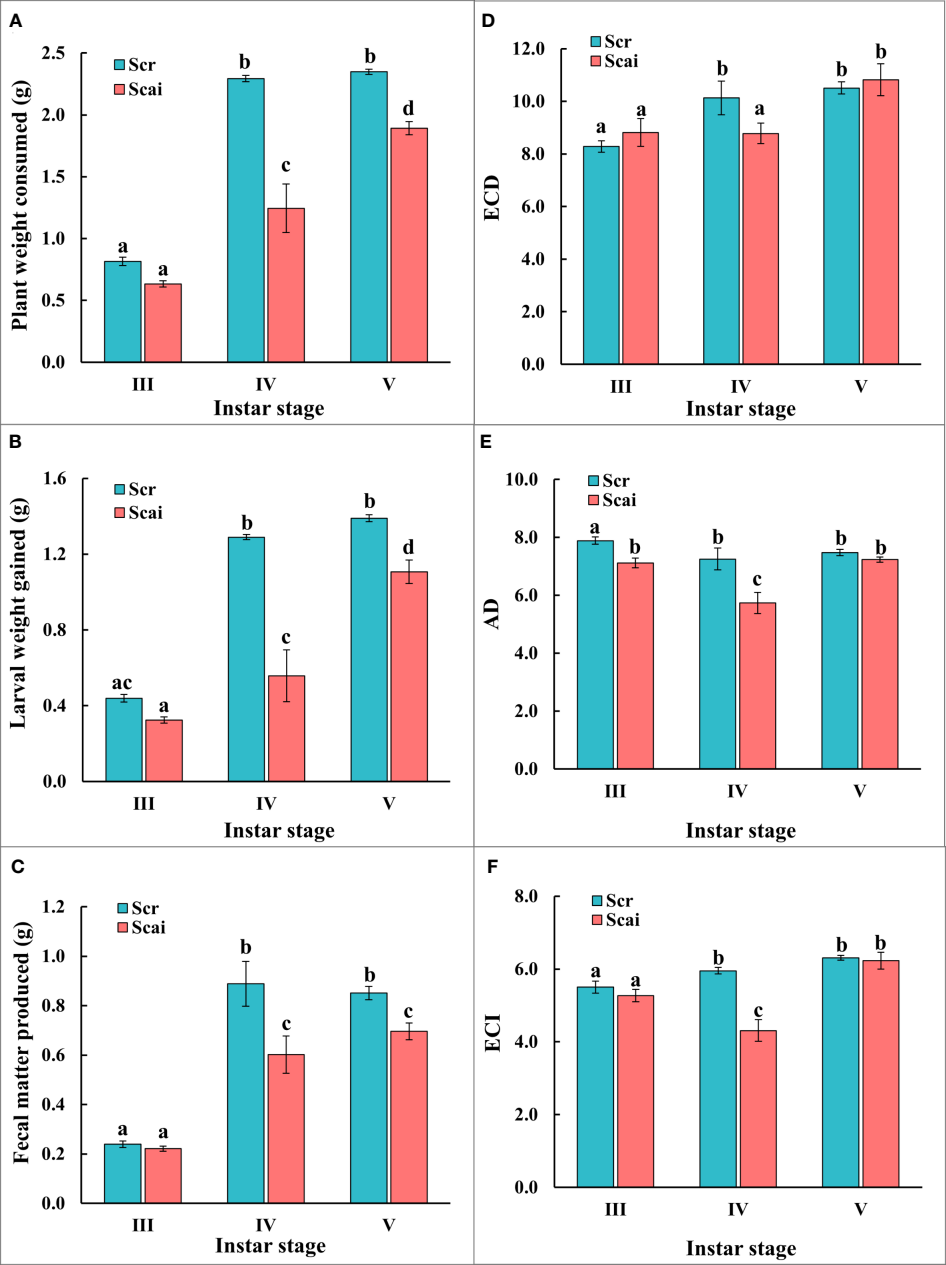
No-choice fixed-time feeding assays were carried out to determine the nutritional indices of third (III), fourth (IV), and fifth (V) instars reared on a Scr diet (blue bars) and Scai diet (red bars). The parameters compared were **(A)** leaf tissues consumed (PWC, g); **(B)** larval weight gain (LWG, g); **(C)** fecal matter produced (FMP, g); **(D)** efficiency of conversion of digested food, ECD; **(E)** approximate digestibility, AD; and **(F)** efficiency of conversion of ingested food, ECI. Bars depict mean ± SE. Significant differences at a *p*-value ≤ 0.05 are denoted by different letters.

The nutritional indices evaluated for actively feeding instars of *S. ricini* were influenced by the developmental age of larvae and dietary host plant type (**Figure 2**). **Figure 2A** shows that larvae from the fourth and fifth instars feeding on *A. excelsa* (Scai) consumed significantly less plant matter (*p* ≤ 0.05) than those of the same age that fed on *R. communis* (Scr). Scr larvae ate similar amounts of plant matter during the fourth and fifth instars, but Scai larvae consumed more *A. excelsa* leaves in the fifth instar than during the fourth instar. **Figure 2B** shows that larvae feeding on Scai gained significantly less weight (*p* ≤ 0.05) than those feeding on Scr during their fourth and fifth instars. **Figure 2C** shows that the amount of fecal matter egested by fourth instars and fifth instars fed on Scai diet was significantly less (*p* ≤ 0.05) than that egested by fourth instar and fifth instars fed Scr diet. The estimates for ECI and ECD showed similar trends in these no-choice fixed-time feeding assays (**Figures 2D, F**), and larvae from fifth instars feeding on Scr and Scai diets had similar values for ECI and ECD. In contrast, lower estimates of ECI and ECD were evident for fourth instars feeding on Scai than those feeding on Scr. The estimates of AD were also significantly lower for fourth instars fed the Scai diet than for larvae fed the Scr diet (**Figure 2E**) indicating that the ingested A. excelsa tissues were digested poorly. Hence, the molecular analyses of genes encoding putative midgut digestive proteases were conducted with fourth instars feeding on *R. communis* and *A. excelsa.*

**Figure S1.1** shows results from a control experiment in which nutritional indices were evaluated for *S. ricini* larvae reared *continuously* on only *R. communis* (Scr) or *A. excelsa* (Scai). The neonates were obtained from the eggs of female moths reared as larvae on a diet of *R. communis*. The results showed that the total weight gained by larvae during development from the neonatal stage to the end of the fifth instar (marked by cessation of feeding, initiation of silk spinning, and cocoon formation) were significantly different (*p* ≤ 0.05), with Scr larvae gaining more weight than Scai larvae (**Figure S1.1A**). Equal amounts of plant tissue were consumed (**Figure S1.1B**), and equal amounts of fecal matter were produced by larvae fed the two plant diets (**Figure S1.1C**). Accordingly, the measures of indices, such as ECI (**Figure S1.1D**) and AD (**Figure S1.1E**), were significantly lower in Scai larvae than in Scr larvae (*p* ≤ 0.05). However, ECD (**Figure S1.1F**) was similar for larvae continuously fed diets of either *R. communis* or *A. excelsa*. **Figure S1.1G** also shows that the relative growth rate of larvae reared continuously on *A. excelsa* alone was significantly lower (*p* ≤ 0.05) than larvae reared on *R. communis* alone, suggesting that diet substantially influenced the growth and development of *S. ricini*.

### Detection of multiple serine proteases in *S. ricini* larval instars and enhanced midgut trypsin activities in larvae transferred to *A. excelsa*

**Figure 3:**
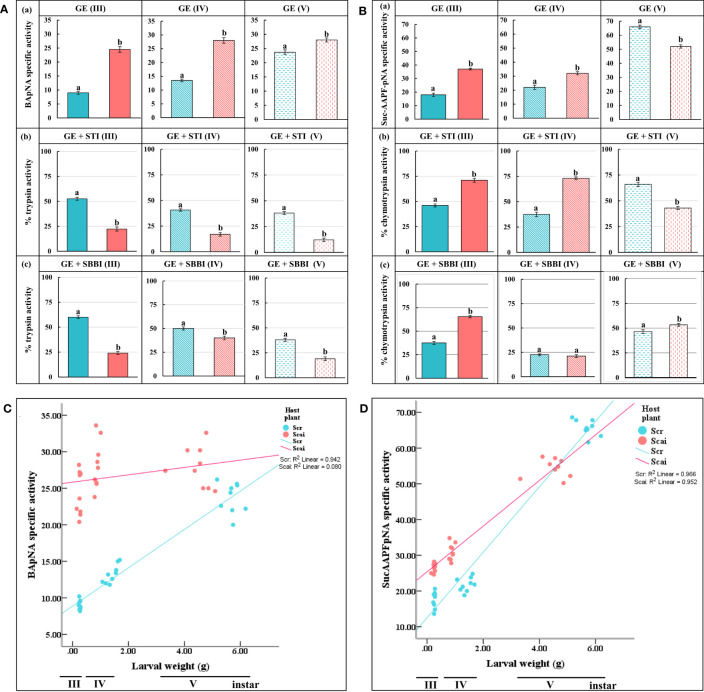
*In vitro* assays for midgut **(A)** trypsin and **(B)** chymotrypsin activities detected per minute per mg total protein in (a) gut extracts (GE) of third (III)-, fourth (IV)-, and fifth (V)-instar *Samia ricini* larvae fed on Scr and Scai diets. The amidolytic substrates used were BApNA for trypsins and SucAAPFpNA for chymotrypsins along with two plant protease inhibitors, (b) STI and (c) SBBI. Bars depict mean ± SE. Significant differences at a *p*-value ≤ 0.05 are denoted by different letters. Percent mean trypsin and chymotrypsin activities detected after inhibition by various inhibitors are shown with reference to the total proteolytic activity detected in the absence of inhibitors (GE) taken as 100%. Correlations of the larval weights of third (III), fourth (IV), and fifth (V) instars of *S. ricini* fed on Scr and Scai diets with **(C)** midgut trypsin activities and **(D)** midgut chymotrypsin activities are shown. Kendall tau coefficients (*R* ^2^) are provided.

Midgut trypsin activity measured at pH 10 was significantly higher (*p* ≤ 0.05) in third, fourth, and fifth instars feeding on *A. excelsa* (Scai) than with instars at the same three stages feeding on *R. communis* (Scr), indicating that protease overproduction was a mode of dietary adaptation in *S. ricini* (**Figure 3Aa**). In Scr samples, we observed that STI inhibited 47.44% of midgut trypsin activity in third instars, 59.32% in fourth instars, and 61.87% in fifth instars (**Figure 3Ab**). The inhibition percentages of midgut trypsin activities by STI in samples from larvae feeding on Scai were 77.78% in third instars, 83.05% in fourth instars, and 87.78% in fifth instars, indicating the upregulation of STI-sensitive trypsins in these larvae. Similar trends were observed for SBBI (**Figure 3Ac**), except that trypsins detected in fourth instars feeding on Scai diets were the least susceptible to SBBI among these developmental stages. The overall results indicated that the nature and properties of midgut trypsins differed with larval age and diet. In addition, hightrypsin activities (> 75% of which were susceptible to STI), were observed in larvae feeding on *A. excelsa*. STI inhibited approximately 50% of the trypsin activities detected in larvae feeding on *R. communis*.

Midgut chymotrypsin activities also increased with larval age, irrespective of diet (**Figure 3Ba**). Chymotrypsin levels were significantly elevated in third (III) and fourth (IV) instars feeding on the Scai diet. However, in fifth (V) instars, midgut chymotrypsin activities were significantly lower in larvae fed the Scai diet than those fed the Scr diet. In contrast to midgut trypsins, midgut chymotrypsin activities in larvae fed the Scai diet were less susceptible to inhibition by STI than those fed the Scr diet (**Figure 3Bb**). The inhibition percentages of midgut chymotrypsins by SBBI in of third, fourth and fifth instars fed the Scr diets were 62.47%, 77.5%, and 53.53%, respectively, whereas SBBI inhibited chymotrypsins in third, fourth, and fifth instars fed the Scai diet by 34.62%, 78.88%, and 46.7%, respectively (**Figure 3Bc**). Interestingly, >75% of chymotrypsin activities detected in fourth-instar *S. ricini* on both diets were inhibited by SBBI. Thus, heterologous protease inhibitors, such as STI and SBBI, inhibited larval gut serine proteases detected in *S. ricini* larvae feeding on *R. communis* and *A. excelsa*, to different extents. A significant and positive correlation was observed in terms of the weight gained by third, fourth, and fifth instars with midgut trypsin activities (**Figure 3C**) detected in larvae fed *R. communis* (*R* ^2^ = 0.942), but no significant correlation was observed for those fed *A. excelsa* (*R* ^2^ = 0.08) (**Figure 3C**). In contrast, larval weight gained in all instars was significant and positively correlated with midgut chymotrypsin activities (**Figure 3D**) in both larvae fed the Scr diet (*R* ^2^ = 0.966) and those fed the Scai diet (*R* ^2^ = 0.952).

### Gelatinolytic zymograms indicate multiple gut proteases with different susceptibilities to PIs in *S. ricini* larval instars fed on *R. communis* and *A. excelsa*

**Figure 4:**
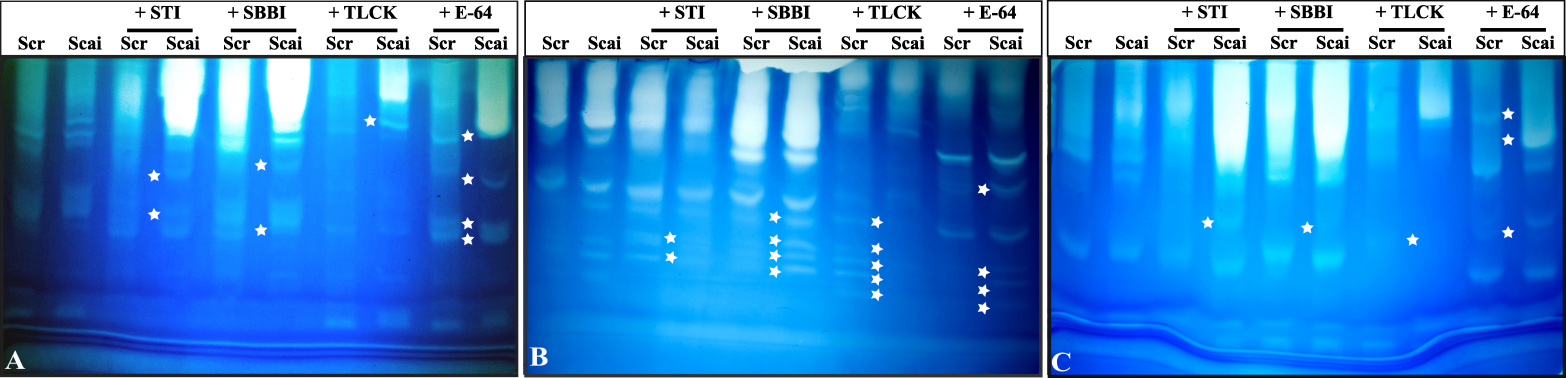
Gelatinolytic zymograms obtained with midgut proteases of **(A)** third, **(B)** fourth, and **(C)** fifth instars of *Samia ricini* reared on *Ricinus communis* (Scr) and *Ailanthus excelsa* (Scai) diets. Differences in activity zones are shown when the midgut samples were incubated with STI, SBBI, TLCK, and E-64 protease inhibitors (1 mg/mL; Sigma-Aldrich; Cat# E3132). Each lane contains equal amounts of total midgut protein. The star-shaped marker denotes a zone of gelatinolytic activity that distinguishes protease complexes (with different mobility and/or inhibitor-susceptibility) detected in gut samples of *S. ricini* larvae feeding on Scr and Scai diets.

Complex profiles with multiple activity zones indicated differences in midgut proteases detected in third, fourth, and fifth instars of *S. ricini* feeding on *R. communis* (Scr) and *A. excelsa* (Scai). The mobilities of fast-, medium- and slow-migrating activity zones were affected upon incubation of gut extracts with diagnostic protease inhibitors such as STI, SBBI, TLCK, and E64, indicating that the abundance and expression of serine proteases in *S. ricini* were influenced by larval age and diet (**Figure 4**). For example, a large number of distinct activity zones were visible for fourth instars feeding on *A. excelsa* (**Figure 4B**). Incubation with STI and TLCK revealed shifts/absence of activity zones in samples from fourth and fifth instars feeding on Scai, indicating the presence of inhibitor-sensitive trypsin activities (**Figures 4B, C**). Activity zones observed in *S. ricini* midgut samples from all three instars were inhibited to a substantial extent by TLCK (**Figures 4A–C**), indicating the prominence of midgut trypsins. These results corroborated trends from the *in vitro* amidolytic assays described earlier (**Figure 3A**).

Multiple midgut proteases were evident in the zymograms of fourth-instar *S. ricini* reared continuously on only *R. communis* (**Figure S2**). A fast-migrating activity zone was absent in samples incubated with STI and SBBI, indicating the presence of inhibitor-sensitive trypsins and/or chymotrypsins (**Figure S2**). The incubation of midgut samples with aprotinin, leupeptin, and E64 resulted in shifts in patterns of slowly migrating activity zones (**Figure S2**). The profiles of activity zones were not affected perceptibly upon incubation with EDTA and pepstatin, indicating that metalloproteinases and acidic hydrolases were not detected under the assay conditions used here.

### Identification of cDNA fragments encoding putative serine proteinase in fourth-instar *S. ricini* feeding on *R. communis* and *A. excelsa*

RT-PCR with serine protease-specific primers (**Table 1**, **Figure S3A**) yielded amplicons of the expected size (i.e., approximately 450 bp). The restriction of fragment length polymorphisms using four-base restriction endnucleases confirmed the presence of heterogeneous cDNA fragments within these amplicons (**Figure S3B**). Cloned RT-PCR and 3′ RACE products (**Figure S4**, **Table S1**) encoded diverse serine proteases that could be grouped into “lineages” with closely related lepidopteran orthologs. Sequence analyses confirmed the presence of active site S195 (numbering after bovine chymotrypsinogen) along with D189, G216, and G226 in the encoded specificity pockets of putative trypsins (Sr III, Sr IV, Sr V, Sr VII, and Sr VIII lineages) and S/G189 in putative chymotrypsins (Sr I and Sr II). The sequence motifs characteristic of mammalian serine proteases (48), including D102 of the catalytic triad, GXG142, and cysteine residues (C168-C182 and C191-C220), were conserved among the encoded proteins (**Figure S4**). Various cDNAs encoded unusual proteases (**Figure S4**) resembling invertebrate brachyurins/collagenases (Sr I and Sr II); proteases with long carboxyl terminal extensions or CTEs (Sr II); and fibrinolytic achelase I (Sr IV). The sequence alignments indicated that there were highly homologous lepidopteran orthologs within each lineage (**Figure S4**), which were characterized by the preponderance of arginine residues (at least eight); shared insertions/deletions; and the occurrence of putative structural loop99 in Sr II and loop170/175 in members of Sr III lineages. Members of the Sr IV lineage shared the motif FPG226VNAR. In fibrinolytic *Lonomia* proteases, this motif occurred as an insertion proximal to G148 and a deletion near position 226 (**Figure S4**). These results suggested that some structure–function relationships of lepidopteran serine proteases were likely to be shared within members of a lineage, but were distinct between lineages. As expected, sequences from this study showed significant homology with contigs of the *S. ricini* genome (#PRJDB9376/GCA_014132275.1; 2020). Some cDNAs matched sequences of intron-less putative chymotrypsin gene fragments cloned from the genomic DNA of *S. ricini* ([(#AFV91961 and #AFV91962; 43)]. Another cDNA corresponded to a putative trypsin gene fragment (#KY67288, Sr VIII lineage), which contained two introns at positions conserved with introns from other insect serine protease genes. Taken together, these results confirmed the presence of a large gene family encoding various serine proteases in *S. ricini*.

### Analysis of multiple sequence lineages encoding diverse serine proteinases in *S. ricini*

**Figure 5:**
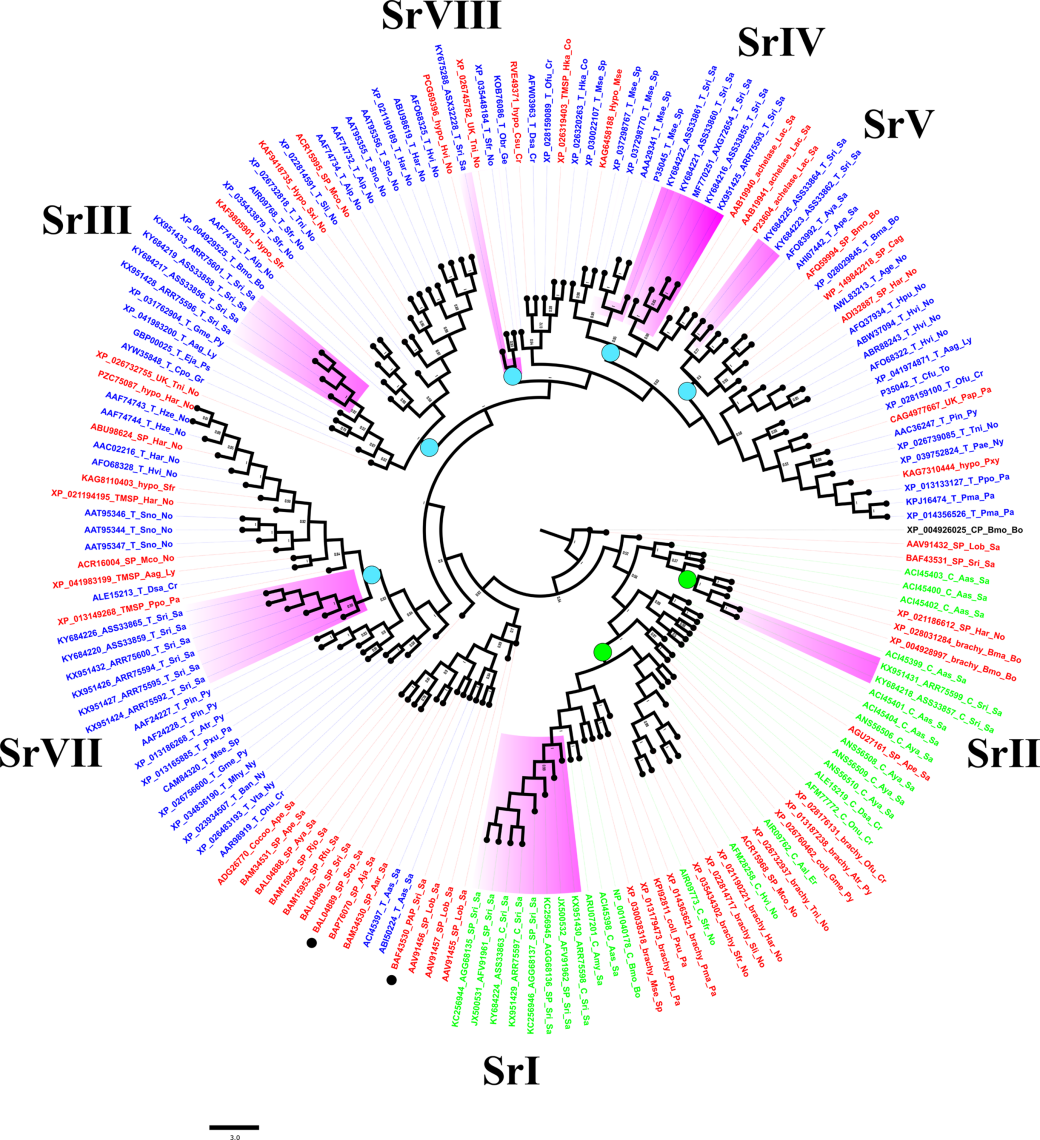
A Bayesian tree based on serine proteases of *Samia ricini* and closely related homologs identified from BLAST searches (**Table S1**). Labels for sequences annotated in NCBI as putative trypsins (T), chymotrypsins (C), and/or serine proteases (SP) are blue, green, and red, respectively. Clades containing *S. ricini* sequences from this study are highlighted in purple. Each label contains an accession number, type of protease, and abbreviated insect and family names. Green and blue circles on various nodes represent sequence lineages with members sharing ≥60% sequence similarity. Bayesian posterior probability values ≥0.5 are shown. Digestive cysteine protease from *Bombyx mori* (GenBank accession# XP_004926025) was used as the outgroup.

A Bayesian tree constructed with 161 closely related lepidopteran serine proteases (**Figure 5**) corroborated results of BLAST-based orthology (**Table S1**). Two main clades bore sequences encoding putative chymotrypsins and trypsins (**Figure 5**, green and blue labels, respectively). The sequences from Sr I and Sr II groups resembled lepidopteran orthologs, including those annotated as brachyurins and/or collagenases after eponymous proteases from crustaceans (**Figure S4**). The Sr I clade included orthologs expressed mostly in the guts of larvae from the families Saturniidae, Noctuidae, Papilionidae, Pyralidae, Sphingidae, Crambidae, and Erabidae, suggesting an ancestral lineage that resembled crustacean digestive brachyurins/collagenases (**Figure S4**). The Sr II clade included orthologs from *B. mori, B. mandarina*, and *H. armigera*, which shared a long semiconserved C-terminal extension rich in clusters of hydrophobic and positively charged amino acids (**Figure S4**). Putative trypsins from this study could be grouped into five clusters (**Figure 5**). Sequences from the Sr III clade were clustered with orthologs from the families Pyralidae, Lycaenidae, Psychidae, and Gracillariidae, and eight species of the Noctuidae family (**Figure 5**, **Figure S4**, **Table S1**). The *S. ricini* Sr IV clade encoded serine proteases that resembled fibrinolytic achelase with plasmin-like activity (UniProt P23604) first reported for the saturniid *L. achelous.* This clade also contained trypsins encoded by a paralogous sequence expansion in *Manduca sexta*, including the first reported lepidopteran gut trypsin (#P35045). The Sr V clade contained putative trypsins from saturniids, such as *A. yamamai* (#JX046916), *A. pernyi* (#KF779933), and various bombycids. These Sr V and Sr VII clusters resembled putative trypsins mostly expressed in the gut from various Lepidoptera families, namely Noctuidae, Lycaenidae, Crambidae, Pyralidae, Papilionidae, Nymphalidae, and Totricidae. The Sr VIII lineage contained a putative trypsin gene fragment from *S. ricini* that resembled alkaline midgut trypsins and/or CFT1-like orthologs predicted from the genomes of several Lepidoptera. **Figure 5** also shows a non-gut trypsin/serine protease-like clade (i.e., not from this study) with sequences from various saturniids (**Figure 5**, black dots) implicated in immunity (#BAF43530, *S. ricini*) and male reproduction (#BAL04889, *S. cynthia pryeri*). In **Figure 5**, the nucleotide and protein sequence similarity in the core regions (spanning H57 to S195) varied between 78% and 99% within each *S. ricini* chymotrypsin lineage, whereas different lineages were only 31%–35% similar. For trypsins, the sequences of core regions within a lineage were at least >65% similar, but among lineages the sequence similarity varied widely, from 28% to 65%. In general, no single cluster was observed in **Figure 5** that contained sequences from *S. ricini* larvae fed on any one host plant.

### Differential gene expression of midgut serine proteinases in *S. ricini* reared on two host plants

**Figure 6:**
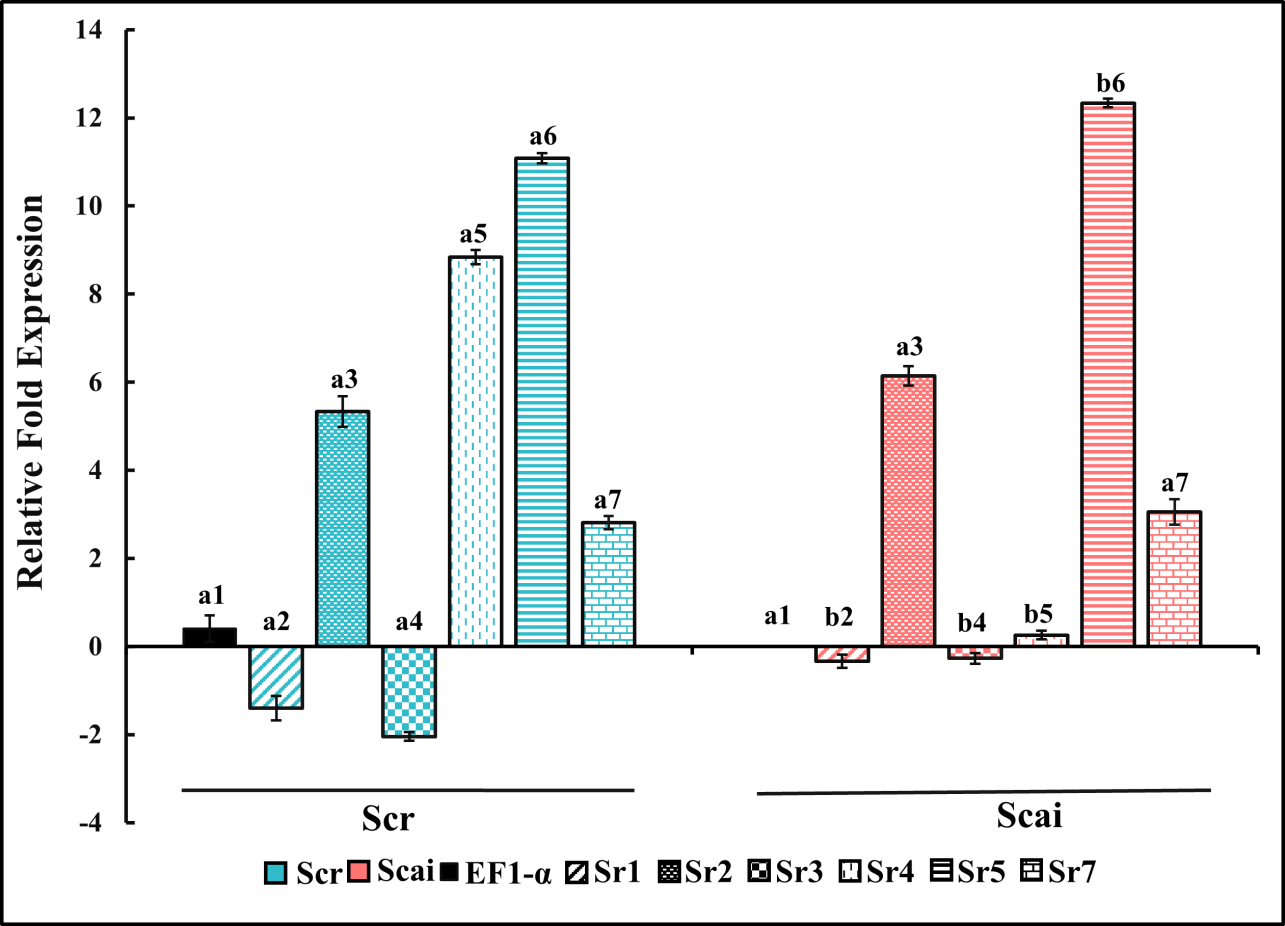
Relative fold gene expression of putative midgut chymotrypsins (Sr1, Sr2) and putative midgut trypsins (Sr3, Sr4, Sr5, Sr7) in fourth-instar *Samia ricini* reared on *Ricinus communis* (Scr) and *Ailanthus excelsa* (Scai) diets. The colors of the histograms for larvae reared on the Scr diet and Scai diet are blue and red, respectively. Decorations within histograms for various genes are as follows: Sr1—diagonal stripes; Sr2—speckles; Sr3—filled dots; Sr4—vertical dashes; Sr5—horizontal stripes; and Sr7—bricks. The data were normalized with respect to levels of elongation factor 1 alpha gene *(EF1-α*) transcripts and GenBank accession #KX951450 from Scai samples (black bar). Bars depicting the standard error are also shown.

**Figure 6** shows the relative fold expression of transcripts amplified by Sr1FP/RP, Sr2FP/RP, Sr3FP/RP, Sr4FP/RP, Sr5FP/RP, Sr7FP/RP, and *EF1-α* primer pairs from midgut RNAs in fourth-instar *S. ricini* feeding on *R. communis* (Scr) and *A. excelsa* (Scai). The gene expression patterns observed included upregulation, downregulation, and there being no change in transcript levels from various lineages. The levels of transcripts amplified by the Sr4FP/RP primer pair, encoding a putative trypsin/achelase (from the Sr IV lineage), showed dramatic differences, with significant upregulation (*p* ≤ 0.01) in larvae feeding on *R. communis* (**Figure 6**, blue vertical striped bar). These mRNAs were downregulated by approximately eightfold in the midguts of larvae feeding on *A. excelsa*, indicating the effect of ingested host type on the expressions of midgut trypsin genes. Transcripts encoding putative trypsins amplified with Sr5FP/RP (from Sr V lineage) were upregulated in the Scai midgut samples in comparison with Scr samples. Transcripts encoding putative chymotrypsins amplified by Sr1FP/RP (from Sr I lineage) and trypsins amplified by Sr3FP/RP (from Sr III lineage) were differentially expressed in two samples, but at levels that were below that detected for the household gene *EF1-α* in Scai samples. Transcripts amplified by the primer pairs Sr2FP/RP (chymotrypsins resembling brachyurins/collagenases from lineage Sr II) and trypsins amplified by SR7FP/RP (from the Sr VII lineage) were expressed at levels approximately fivefold higher than that of the household gene *EF1-α* in Scai larvae. However, there were no statistical differences between their levels in the midgut samples of larvae fed on Scr and Scai diets. Taken together, these results indicated the differential expression of several cDNAs and *status quo* for the levels of some transcripts encoding midgut serine proteinases in *S. ricini* larvae feeding on the two species of host plants.

## Discussion

Comparative life-history studies generally reaffirm that the utilization of particular host plant types with characteristic defenses influence growth, development, and, ultimately, complex traits such as the fitness of phytophagous lepidopteran insects (9, 38, 49–52). In this study, *R. communis* was a better host plant than *A. excelsa*, as *S. ricini* larvae fed on the Scr diet displayed significantly higher (*p* ≤ 0.05) larval weight gain, cocoon shell weight, realized fecundity, shorter larval periods, and lower mortality than those fed on Scai diets (**Figure 1**). Comparable estimates have been reported for life-history parameters of *S. ricini* reared on *R. communis* (36, 53–55). The superior performance for various life-history traits in larvae fed Scr diets most probably reflects dietary habituation as a result of domestication on *R. communis* plants. Further work is necessary to determine factors in the ancestral host *A. excelsa* that reduce the performance of *S. ricini*, especially for sericulture-related traits, namely the low weights of larvae, pupae, and cocoons, and low-realized fecundity (**Figure 1**, **Figure S1.1**). Although larvae reared on *R. communis* performed better for most life-history traits than those reared on *A. excelsa*, larvae reared on *A. excelsa* nevertheless showed ≥90% survival rates, produced cocoons, and successfully completed their life cycles (**Figure 1**; **Figure S1.2**). The weights of fifth-instar larvae and pupae obtained from insects reared on Scr diets had a higher median value than those of insects reared on Scai diets (**Figure S1.3**), but insects from both diet groups showed 100% eclosion. The mortality of insects reared on Scai diets was observed only in early instars, and all larvae that reached fourth instars survived into adulthood. These results indicated that *A. excelsa* was a viable alternative host for rearing *S. ricini* larvae, as dietary acceptance and ability to complete the life cycle are important determinants of host plant utilization by polyphagous insects (23, 56).

The estimation of nutritional indices can provide valuable information on the physiological aspects of insect–plant interactions (37, 38, 52). The effects of varied host plant choice on nutritional indices have been shown for highly polyphagous Lepidoptera, such as *Antheraea polyphemus* (57), *Helicoverpa armigera* (58), *Spodoptera litura* (59), and *S. ricini* (9, 60). In this study, no-choice fixed-time feeding assays showed that the ECD index was significantly lower in fourth-instar *S. ricini* larvae transferred to the Scai diet than those fed on the Scr diet (**Figure 2D**). This decrease in the ECD index has been attributed to an increase in insect metabolism for the maintenance of physiological functions or detoxification of ingested plant secondary defense compounds (37, 61). Similar results have been found for *S. ricini* reared on *A. excelsa, A. altissima*, and *R. communis* (36, 53, 54, 62). Furthermore, the AD index was also lower in third- and fourth-instar *S. ricini* fed on the Scai diet than those fed on the Scr diet (**Figure 2E**), suggesting that dietary constituents, such as fiber contents, antifeedants, and secondary metabolites, which can differentially influence digestion in *S ricini* larvae feeding on the two plant species, need to be identified. Interestingly, the ECI and ECD indices were similar in fifth-instar *S. ricini* fed on both diets, suggesting that metabolic dexterity improves with larval age (**Figure 2**). Improved metabolism has also been reported in older instars of pests such as *Lymantria dispar, Ostrinia nubilalis*, *Helicoverpa zea*, and *H. armigera* (63–65).

Proteolysis by serine proteases is an important component of the overall digestion of ingested food in lepidopteran larvae. It contributes nutrients, such as short peptides and free amino acids, which are essential for optimal growth and development (17). Larval weight gain had a poor correlation with changes in midgut trypsin levels in larvae feeding on *A. excelsa*, but not in those feeding on *R. communis* (**Figure 3C**), suggesting that trypsin overproduction may extract metabolic costs and negatively influence larval growth in larvae feeding on a Scai diet. Midgut serine proteases, especially trypsin and chymotrypsin activities detected in *S. ricini* larval instars feeding on the two diet types, showed different susceptibilities to STI (**Figure 3Ab**; **Figure 3Bb**). Older-instar larvae fed on the Scai diet had significantly higher levels of STI-susceptible midgut trypsin activities than those larvae fed on the Scr diet (**Figure 3Ab**). A similar increase in gut serine proteinase activities, and a change in protease sensitivity to inhibitors are classic indicators of the physiological adaptation of lepidopteran larvae to dietary PPI (66, 67). The metabolic costs incurred in the adaptive responses to dietary changes involving the differential expression of digestive serine proteases can be manifested as poor growth and fitness of the larvae (39, 68–70). Trypsin-inhibitory activities, which need to be characterized further, were detected in different leaf types of *R. communis* and *A. excelsa* (43). Trypsin inhibitory activities have also been reported in crude leaf extracts and a lipid fraction of the castor seed cake in *R. communis* (71). As the pupae of *S. ricini* are edible (72), it would be of some interest to trace the fate of ingested antifeedants, such as PPI, that influence larval adaptation to different host plants. The breeding of host plants with reduced levels of foliar protease inhibitors could also be attempted for the improved rearing of commercial stocks of *S. ricini*.

Large gene families encoding diverse serine proteases from clan PA, family S1 (E.C. 3.4.21.1) with tryptic, chymotryptic, and other unknown specificities are expressed in the larval guts of phytophagous Lepidoptera. At least 38 serine proteases belonging to distinct groups/lineages are reported from the midgut of *B. mori* (18). The groups include paralogs/isoforms that share high sequence homology, and some represent species-specific expansions within the gene family that enable the silkworm to digest mulberry leaves (18). In this study, we report on lineages within a large serine protease gene family in *S. ricini* that encode midgut enzymes with distinct sequence features (**Figure S4**) and share common ancestry with orthologous serine proteases from various Lepidoptera families (**Figure 5**). In contrast to invertebrate and mammalian enzymes, the lepidopteran enzymes in various lineages contained at least eight arginine residues (**Figure S4**) that may be related to the alkaline midgut environment (73). The cDNAs identified in *S. ricini* with the DmTF/SerPR primer pair and related lepidopteran orthologs (Sr I and II lineages; **Table S1**; **Figure S4**) encoded proteases that resembled brachyurin-C/brachyurin Ia-Ib expressed in guts of crustacean brachypods [EC 3.4.21.32; (74)]. The sequence similarity with invertebrate serine proteases suggested that these members of the serine protease gene family, which were transcribed in the gut tissues of larval *S. ricini*, were of ancient origin and probably predated the domestication process of *S. ricini*. The crustacean proteases are typically detected with peptidyl substrates, such as SAAPFpNA (as used in this study), and display the wide substrate specificity necessary for the digestion of dietary type I collagen (74). The functional implications of such structural features in lepidopteran proteases are currently unclear. The crustacean enzymes show replacements of S189G and G226D (when compared with bovine chymotrypsinogen) that can influence the conformation of their substrate specificity pockets (75). **Figure S4** showed that all lepidopteran orthologs in the Sr I clade contained the S189G substitution but showed a G226S substitution that probably rendered the S1 pocket of encoded enzymes more polar. In the case of the Sr II lineage, three Glu residues and a short look around position 99 were observed (**Figure S4**). These structural features are known to impact substrate specificities in serine proteases (76). Members of the *S. ricini* Sr II lineage also contained a characteristic CTE with the embedded motif “ELLKRLEVKVKVKVK” (**Figure S4**) of unknown function.

A significant increase in midgut trypsin activities in larvae fed on Scai diets (**Figure 3Aa**) supported the idea that these enzymes play a prominent role in digestion. Transcripts encoding multiple different putative trypsins belonging to distinct sequence lineages were observed in *S. ricini* larval guts (**Figure 5**). Some of these cDNAs were also expressed differently in response to diet (**Figure 6**). **Table S1** shows that many lepidopteran orthologs from these lineages are associated with the physiological adaptations of larvae to dietary changes and the ingestion of PPIs. For example, the cDNAs from the Sr III lineage resembled the AiT6 and AiT9 cDNAs from *Agrotis ipsilon* fourth instars upregulated on artificial diets containing 1% STI (45). Members of the Sr III lineage shared an insertion around position 170/175, which forms a putative loop associated with allosteric network interactions between functionally relevant residues in mammalian trypsins (76). The Sr IV lineage resembled fibrinolytic achelases from venomous saturniids, such as *L. achelous* (77), that degrade complex substrates such as cocoons, fibrins, and fibrinogens. Reasons for the dramatic differential expression of these transcripts in *S. ricini* larvae feeding on the Scai diet requires further investigation. The Sr V clade contained various paralogs from *M. sexta*, of which at least one is a putative alkaline trypsin expressed in the gut (73), whereas an ortholog (ApTLS) is a trypsin upregulated in response to infection with *B. mori* by the microsporidian pathogen *Nosema* (30). The members of the Sr VII lineage resembled gut proteases from *Diatraea saccharalis*, *Plodia interpunctella*, and *Plutella xylostella*, which are known to be differentially expressed in larvae feeding on potential antifeedants, such as PPIs and Bt endotoxins (78–80).

According to Lopes et al. (14), midgut serine proteases can be grouped into two groups: PPI sensitive and PPI insensitive. Sequence alignments reported from Lepidoptera indicate that position 190 is typically a Gln in PI-sensitive trypsins and an Ala in PI-insensitive trypsins (14, 79). There is some support in the literature for larval growth of Spodoptera frugiperda and D. saccharalis (79) where such clear demarcations are evident. However, more generally, processes of dietary adaptations tend to be complex, as lepidopteran larvae can encounter multiple and/or novel PPIs that vary in quantity or quality, reflecting the physiological changes in phenology of different host plant types (31, 81). In this study, the serine proteases mostly contained Q190, except for Sr III and Sr VII, which contained A190, suggesting that these midgut proteases in *S. ricini* probably have different susceptibilities to PPIs. The ability to regulate the expression of PPI-sensitive/-insensitive serine protease genes in gut tissues may enable larvae to utilize and adapt to multiple host plants from different taxonomic families containing characteristic PPIs. Serine proteases encoded by different sequence lineages were likely to have distinct structures, nuanced substrate specificities, and protease inhibitor interactions. The plasticity of proteolysis and/or their inhibition probably influenced related functions in the larval gut, such as the digestion of plant proteins, zymogen activation, immunity, and development. The processes for co-opting particular member(s) of lineage(s) encoding midgut serine proteases during utilization and/or adaptation to a particular host plant type are not immediately apparent.

The process of domestication in *B. mori* has been under active investigation (82–84). In a comparison of genomes of 40 silkworms to identify loci segregating with domestication events, several serine proteinases expressed in the midgut and involved in digestion were identified (82). In the case of *S. ricini*, domestication by selection on *R. communis* as the larval host plant has not made this polyphagous insect into a monophagous one. It has, however, enhanced its performance when it is reared on a diet of *R. communis.* This study identified diverse lineages of genes encoding midgut serine proteinases in the domesticated polyphagous *S. ricini*, and demonstrated the differential expression of some proteases, especially midgut trypsins, produced in larvae feeding on *R. communis* (the host plant implicated in domestication) and transferred to *A. excelsa* (an ancestral host tree). Such information may be useful for the examination of domestication events that tamed *S. ricini* and relevant for the domestication of economically important saturniids, such as *A. assamensis, A. pernyi*, and *Antheraea mylitta*, which are currently reared on trees in plantations and forests. The domestication of these silkmoths would enable the conservation of wild populations, reduce cocoon collection from forests, and preserve valuable gene pools for useful traits, such as disease resistance (85), benefiting the tribal peoples and indigenous communities who depend on these cocoon silks for alternative livelihoods.

It is currently unclear whether multiple generations of selection and continuous rearing will produce populations of *S. ricini* habituated to a newer host plant type (albeit an ancestral food plant). In this study, neonates (F1) originating from and reared on Scai diets showed lower estimates for nutritional indices, relative growth rate, and percentage survival than larvae reared continuously on Scr diets (**Figure S1**), probably reflecting post-ingestive metabolic costs. Similar results have been reported for highly polyphagous larvae of *H. armigera*, which do not show improved performance on a “less optimal” host even after multiple rounds of selection (65). Therefore, our results suggest that dietary habituation may occur at a faster rate on mixed diets, namely those on which older larvae are transferred from a *R. communis* diet to an alternative host such as *A. excelsa*. Such dietary changes can also be attempted for “reverse domestication”, for which the older larvae of wild *S. canningi* and/or its hybrids with *S. ricini* are transferred to a new host to obtain populations suitable for commercial rearing. In fact, fertile F1 hybrids are reported among wild *S. canningi* and domesticated *S. ricini* reared on the families Rutaceae and castor (86).

*S. ricini* sericulture (or “ericulture”) typically involve family-run small-cottage enterprises [**Figure S1.4**; (87)]. *A. excelsa* is well suited as a candidate for host tree plantations. *A. excelsa* is a multifunctional tree, rich in the bioactive compounds used in tribal medicine, and an important source of fiber for ruminant feed (88). However, improving the larval performance of *S. ricini* on *A. excelsa* for commercial ericulture and silkworm rearing requires further research. For example, the impact of ingested foliar flavonoids and triterpene derivatives, such as the bitter quassinoids, excelsin, and ailanthinone in *A. excelsa*, with known antifeedant and insect repellent properties (88) on *S. ricini* larvae is not known. Interestingly, the processes enabling prolific utilization of castor leaves containing a panoply of phytochemicals, such as ricin, ricinine, flavonoids, saponins, condensed tannins, quercetins, and phenols (89) by *S. ricini*, also await elucidation. The roles of genes and gene families encoding cytochrome P450 monooxygenases, carboxylesterases, choline esterases, glutathione *S*-transferases, UDP glycosyltransferases, glucose oxidases, and other detoxification strategies identified in polyphagous lepidopteran pests (90, 91) need to be investigated in *S. ricini* larvae. New perspectives for understanding the process of domestication in *S. ricini* on castor are urgently required. Efforts to reutilize ancestral host plants, such as *A. excelsa*, may benefit from studies on microbial communities associated with *S. ricini* larvae feeding on different diets. Recent information suggests that there are dynamic communities of aerobic and anaerobic gut endosymbionts in *S. ricini* larvae feeding on *R. communis* with the potential to utilize complex substrates such as lignocellulose (92, 93). Detailed characterization of the microbial constituents, metagenomes, and functions of the larval “holobiont” (94, 95) in *S. ricini* feeding on various hosts, such as *R. communis* and *A. excelsa*, may pay rich dividends in improving the commercial productivity of this economically important domesticated polyphagous silkworm to the benefit of its indigenous rearers.

## Data availability statement

The datasets presented in this study can be found in online repositories. The names of the repository/repositories and accession number(s) can be found in the article/**Supplementary Material**.

## Author contributions

SK collected research material, designed and performed experiments, analyzed data and wrote the first draft; PB analyzed data, wrote and edited drafts; MS performed experiments; SM-L conceived and supervised the research, obtained grants, analyzed data, wrote and edited drafts. All authors contributed to the article and approved the submitted version.
