# Supplementary material for: Midgut serine proteinases participate in dietary adaptations of the castor (Eri) silkworm Samia ricini Anderson transferred from Ricinus communis to an ancestral host, Ailanthus excelsa Roxb
Source: Front Insect Sci. 2023 Aug 10;3:1169596. doi: 10.3389/finsc.2023.1169596 (PMC10926435; doi:10.3389/finsc.2023.1169596)
Supplement: Supplementary file 1 [file DataSheet_1.zip › Table S1.pdf]

Table S1: Sequences of putative serine proteases used for Figure 5.									
**#**	**Nucleotide ID (Clone)**	**Protein ID**	**Size (aa)**	**Insect**	**Generalist/ Specialist**	**Family**	**Preferred Host/Family**	**Definition**	**Expressed Location**
**1**	**KC256944 (ME19N)**	**AGG68135**	**151**	***Samia ricini***	**Generalist**	**Saturniidae**	***Ricinus communis***	**Serine proteinase mRNA**	**Midgut**
**2**	**JX500531 (ScrE3)**	**AFV91961**	**153**	***Samia ricini***	**Generalist**	**Saturniidae**	***Ricinus communis***	**Putative serine proteinase**	**Whole organism**
**3**	**KY684224 (1AiSO10n)**	**ASS33863**	**154**	***Samia ricini***	**Generalist**	**Saturniidae**	***Ailanthus excelsa***	**Putative chymotrypsin mRNA**	**Midgut**
**4**	**KX951429 (2RcSOR3nRC 1-5)**	**ARR75597**	**198**	***Samia ricini***	**Generalist**	**Saturniidae**	***Ricinus communis***	**Putative chymotrypsin mRNA**	**Midgut**
**5**	**KC256946 (ME19)**	**AGG68137**	**151**	***Samia ricini***	**Generalist**	**Saturniidae**	***Ricinus communis***	**Serine proteinase mRNA**	**Midgut**
**6**	**KC256945 (ME27)**	**AGG68136**	**151**	***Samia ricini***	**Generalist**	**Saturniidae**	***Ricinus communis***	**Serine proteinase mRNA**	**Midgut**
**7**	**JX500532 (ScrE37)**	**AFV91962**	**153**	***Samia ricini***	**Generalist**	**Saturniidae**	***Ricinus communis***	**Putative serine proteinase**	**Whole organism**
**8**	**KX951430 (9RC8RC1-14)**	**ARR75598**	**198**	***Samia ricini***	**Generalist**	**Saturniidae**	***Ricinus communis***	**Putative chymotrypsin mRNA**	**Midgut**
9	KX580883	ARU07201	157	*Antheraea mylitta*	Generalist	Saturniidae	*Terminalia arjuna, Shorea robusta*	chymotrypsin, partial	Whole organism
10	FJ217714	ACI45398	154	*Antheraea assama*	Generalist	Saturniidae	*Persea bombycina*	Putative chymotrypsin mRNA	Midgut
11	NM_001046713	NP_001040178	292	*Bombyx mori*	Specialist	Bombycidae	*Morus alba*	chymotrypsinogen precursor	na
12	XM_030182458	XP_030038318	289	*Manduca sexta*	Specialist	Sphingidae	Solanaceae	brachyurin-like	body, legs, wings, head
13	XM_013324019	XP_013179473	290	*Papilio xuthus*	Specialist	Papilionidae	*Citrus sp.*	brachyurin-like	na
14	KQ459603	KPI92811	290	*Papilio xuthus*	Specialist	Papilionidae	*Citrus sp.*	Collagenase	Whole body

15	XM_014508135	XP_014363621	290	*Papilio machaon*	Specialist	Papilionidae	*Pimpinella saxifrage*	brachyurin-like	na
16	KM083792	AIR09773	289	*Spodoptera frugiperda*	Generalist	Noctuidae	*Hordeum sp.*	Chymotrypsin mRNA	Midgut
17	XM_035578409	XP_035434302	289	*Spodoptera frugiperda*	Generalist	Noctuidae	*Hordeum sp.*	brachyurin-like	Whole body
18	XM_022958949	XP_022814717	289	*Spodoptera litura*	Generalist	Noctuidae	*Gossypium sp., Arachis hypogaea*	brachyurin-like	Whole body
19	XM_021334546	XP_021190221	289	*Helicoverpa armigera*	Generalist	Noctuidae	*Gossypium sp.*	brachyurin-like	Whole body
20	JN252045	AFM28258	289	*Heliothis virescens*	Generalist	Noctuidae	*Nicotiana sp.*	Chymotrypsin mRNA	Midgut
21	FJ205399	ACR15968	280	*Mamestra configurata*	Generalist	Noctuidae	*Canola sp.*	Serine protease mRNA	Midgut
22	XM_026877136	XP_026732937	285	*Trichoplusia ni*	Generalist	Noctuidae	Crucifers	brachyurin-like	ovarian cell line Hi7
23	KM083781	AIR09762	288	*Alabama argillacea*	Specialist	Erebidae	*Gossypium sp.*	chymotrypsin-like serine protease precursor	Midgut
24	XM_026904661	XP_026760462	286	*Galleria mellonella*	Generalist	Pyralidae	*Figus sp.*	Collagenase-like	whole adult
25	XM_013331784	XP_013187238	292	*Amyelois transitella*	Generalist	Pyralidae	Fig, walnut, almond, pistachio	brachyurin-like	na
26	XM_028320330	XP_028176131	292	*Ostrinia furnacalis*	Generalist	Crambidae	*Zea mays*	brachyurin-like	Pupae
27	**KX951431 (8RcOBSR34R CB2-4)**	**ARR75599**	**286**	***Samia ricini***	**Generalist**	**Saturniidae**	***Ricinus communis***	**Putative chymotrypsin mRNA**	**Midgut**
28	**KY684218 (7AiOBS9)**	**ASS33857**	**155**	***Samia ricini***	**Generalist**	**Saturniidae**	***Ailanthus excelsa***	**Putative chymotrypsin mRNA**	**Midgut**
29	FJ217715	ACI45399	155	*Antheraea assama*	Generalist	Saturniidae	*Litsea monopetala*	Putative chymotrypsin mRNA	Midgut

30	XM_004928940	XP_004928997	369	*Bombyx mori*	Specialist	Bombycidae	Morus alba	brachyurin	na
31	XM_028175483	XP_028031284	369	*Bombyx mandarina*	Specialist	Bombycidae	*Morus alba*	brachyurin-like	Silk gland
32	**KX951433 (4RcORR6RC3-4)**	**ARR75601**	**200**	***Samia ricini***	**Generalist**	**Saturniidae**	***Ricinus communis***	**Putative trypsin mRNA**	**Midgut**
33	**KY684219 (3AiOAR7)**	**ASS33858**	**161**	***Samia ricini***	**Generalist**	**Saturniidae**	***Ailanthus excelsa***	**Putative trypsin mRNA**	**Midgut**
34	**KY684217 (4RcORR20)**	**ASS33856**	**161**	***Samia ricini***	**Generalist**	**Saturniidae**	***Ricinus communis***	**Putative trypsin mRNA**	**Midgut**
35	**KX951428 (8RcOBSR4)**	**ARR75596**	**161**	***Samia ricini***	**Generalist**	**Saturniidae**	***Ricinus communis***	**Putative trypsin mRNA**	**Midgut**
36	XM_030176284	XP_031762904	264	*Galleria mellonella*	Generalist	Pyralidae	*Figus sp.*	trypsin, alkaline B-like isoform X2	whole adult
37	XM_042118941	XP_041983200	190	*Aricia agestis*	Generalist	Lycaenidae	*Helianthemum nummulariu,* Geraniaceae	trypsin, alkaline A-like	na
38	BGZK01000004	GBP00025	263	*Eumeta japonica*	Specialist	Psychidae	Fagaceae, Quercus sp.	Trypsin alkaline C	na
39	MG993089	AYW35848	157	*Cameraria pongamiae*	Specialist	Gracillaridae	*Millettia pinnata*	putative trypsin, partial	gDNA
40	XM_026876954	XP_026732755	754	*Trichoplusia ni*	Generalist	Noctuidae	Crucifers	uncharacterized protein LOC113497409	ovarian cell line Hi5
41	XM_004929468	XP_004929525	263	*Bombyx mori*	Specialist	Bombycidae	Morus alba	trypsin, alkaline C	na
42	AF261971	AAF74733	262	*Agrotis ipsilon*	Generalist	Noctuidae	Poaceae	Trypsin precursor mRNA	Midgut
43	JACKWZ010000051	KAF9805901	266	*Spodoptera frugiperda*	Generalist	Noctuidae	Poaceae, Asteraceae, Fabaceae	hypothetical protein SFRURICE_013877	Whole body, pupa
44	XM_035577994	XP_035433879	266	*Spodoptera frugiperda*	Generalist	Noctuidae	*Hordeum sp.*	trypsin, alkaline C-like	Whole body
45	KM083787	AIR09768	266	*Spodoptera frugiperda*	Generalist	Noctuidae	*Hordeum sp.*	trypsin-like serine protease precursor	Midgut

46	XM_026877017	XP_026732818	281	*Trichoplusia ni*	Generalist	Noctuidae	Crucifers	trypsin, alkaline C-like	ovarian cell line Hi6
47	JACKWZ010000051	KAF9418735	263	*Spodoptera exigua*	Generalist	Noctuidae	*Allium fistulosum*	hypothetical protein HW555_004563	Whole body, pupa
48	XM_022958949	XP_022814591	263	*Spodoptera litura*	Generalist	Noctuidae	*Gossypium sp., Arachis hypogaea*	trypsin, alkaline C-like	Whole body
49	FJ205428	ACR15995	263	*Mamestra configurata*	Generalist	Noctuidae	*Canola sp.*	serine protease 5	Midgut
50	AF261972	AAF74734	202	*Agrotis ipsilon*	Generalist	Noctuidae	Poaceae	Trypsin precursor mRNA	Midgut
51	AF261970	AAF74732	263	*Agrotis ipsilon*	Generalist	Noctuidae	Poaceae	Trypsin precursor mRNA	Midgut
52	AY587163	AAT95355	223	*Sesamia nonagrioides*	Generalist	Noctuidae	*Zea mays*	trypsin III precursor	Midgut
53	AY587164	AAT95356	263	*Sesamia nonagrioides*	Generalist	Noctuidae	*Zea mays*	trypsin III precursor	Midgut
54	EF600054	ABU98619	263	*Helicoverpa armigera*	Generalist	Noctuidae	*Gossypium sp.*	Protease mRNA	Gut
55	XM_021334514	XP_021190189	263	*Helicoverpa armigera*	Generalist	Noctuidae	*Gossypium sp.*	trypsin CFT-1-like	Whole body
56	JN793545	AFO68325	263	*Heliothis virescens*	Generalist	Noctuidae	*Nicotiana sp.*	Trypsin mRNA	Midgut
57	NWSH01002022	PCG69396	263	*Heliothis virescens*	Generalist	Noctuidae	*Nicotiana sp.*	hypothetical protein B5V51_4161	na
58	**KY684222 (7AiOBS15)**	**ASS33861**	**102**	***Samia ricini***	**Generalist**	**Saturniidae**	***Ailanthus excelsa***	**Putative trypsin mRNA**	**Midgut**
59	**KY684221 (10Ai14)**	**ASS33860**	**141**	***Samia ricini***	**Generalist**	**Saturniidae**	***Ricinus communis***	**putative trypsin, partial**	**Midgut**
60	**MF770251 (Ai14)**	**AXG72654**	**193**	***Samia ricini***	**Generalist**	**Saturniidae**	***Ricinus communis***	**putative trypsin, partial**	**Midgut**
61	**KY684216 (6RcORC33)**	**ASS33855**	**154**	***Samia ricini***	**Generalist**	**Saturniidae**	***Ricinus communis***	**Putative trypsin mRNA**	**Midgut**
62	**KX951425 (6RcORC4)**	**ARR75593**	**150**	***Samia ricini***	**Generalist**	**Saturniidae**	***Ricinus communis***	**Putative trypsin mRNA**	**Midgut**

63	AAB19940	AAB19940	214	*Lonomia achelous*	Generalist	Saturniidae	*Tapirira guianensis*	achelase II=fibrinolytic proteinase	Saliva/Hemolymph
64	AAB19941	AAB19941	213	*Lonomia achelous*	Generalist	Saturniidae	*Tapirira guianensis*	achelase I=fibrinolytic proteinase	Saliva/Hemolymph
65	P23604	P23604	213	*Lonomia achelous*	Generalist	Saturniidae	*Tapirira guianensis*	Achelase-I	na
66	XM_030166247	XP_030022107	256	*Manduca sexta*	Specialist	Sphingidae	Solanaceae	trypsin, alkaline C-like	body, legs, wings, head
67	JACWZX010000488	KAG6458188	648	*Spodoptera frugiperda*	Generalist	Noctuidae	Poaceae, Asteraceae, Fabaceae	hypothetical protein SFRURICE_013853	Whole body
68	XM_035597206	XP_037298767	262	*Manduca sexta*	Specialist	Sphingidae	Solanaceae	trypsin, alkaline A-like	body, legs, wings, head
69	XM_037442876	XP_037298770	256	*Manduca sexta*	Specialist	Sphingidae	Solanaceae	trypsin, alkaline A-like	body, legs, wings, head
70	L16807	AAA29341	255	*Manduca sexta*	Specialist	Sphingidae	Solanaceae	Alkaline midgut trypsin mRNA	Midgut
71	P35045	P35045	256	*Manduca sexta*	Specialist	Sphingidae	Solanaceae	Trypsin	na
72	**KY684225 (7AiOBS7)**	**ASS33864**	**163**	***Samia ricini***	**Generalist**	**Saturniidae**	***Ricinus communis***	**Putative trypsin mRNA**	**Midgut**
73	**KY684223 (7AiOBS8)**	**ASS33862**	**108**	***Samia ricini***	**Generalist**	**Saturniidae**	***Ricinus communis***	**Putative trypsin mRNA**	**Midgut**
74	JX046916	AFO83992	265	*Antheraea yamamai*	Generalist	Saturniidae	*Quercus sp.*	Trypsin mRNA	Unknown
75	KF779933	AHI07442	257	*Antheraea pernyi*	Generalist	Saturniidae	*Quercus sp.*	Trypsin mRNA	Fat Body
76	JX312360	AFQ59994	256	*Bombyx mori*	Specialist	Saturniidae	*Morus alba*	alkaliphilic serine protease	na
77	XM_028174044	XP_028029845	256	*Bombyx mandarina*	Specialist	Bombycidae	*Morus alba*	trypsin, alkaline C-like	Silk gland
78	WP_149842218	WP_149842218	255	*Chitinophaga agrisoli*	Bacteria	Chitinophagaceae	na	serine protease, partial	na

79	MF407317	AWL83213	260	*Anticarsia gemmatalis*	Generalist	Noctuidae	*Glycine max*	Trypsin 1	Gut
80	**KY684226 (6RcORC1)**	**ASS33865**	**199**	***Samia ricini***	**Generalist**	**Saturniidae**	***Ricinus communis***	**Putative trypsin mRNA**	**Midgut**
81	**KY684220 (5AiOAC1)**	**ASS33859**	**175**	***Samia ricini***	**Generalist**	**Saturniidae**	***Ailanthus excelsa***	**Putative trypsin mRNA**	**Midgut**
82	**KX951432 (5AiOAC13Ai7-7)**	**ARR75600**	**199**	***Samia ricini***	**Generalist**	**Saturniidae**	***Ailanthus excelsa***	**Putative trypsin mRNA**	**Midgut**
83	**KX951426 (6RCORC13)**	**ARR75594**	**153**	***Samia ricini***	**Generalist**	**Saturniidae**	***Ricinus communis***	**Putative trypsin mRNA**	**Midgut**
84	**KX951427 (6RcORC16)**	**ARR75595**	**153**	***Samia ricini***	**Generalist**	**Saturniidae**	***Ricinus communis***	**Putative trypsin mRNA**	**Midgut**
85	**KX951424 (5AiOAC2)**	**ARR75592**	**158**	***Samia ricini***	**Generalist**	**Saturniidae**	***Ailanthus excelsa***	**Putative trypsin mRNA**	**Midgut**
86	XM_013293814	XP_013149268	508	*Papilio polytes*	Specialist	Papilionidae	*Citrus sp.*	transmembrane protease serine 9-like	na
87	KR024671	ALE15213	261	*Diatraea saccharalis*	Generalist	Crambidae	*Saccharum officinarum*	trypsin-like serine protease precursor	Midgut
88	XM_042118939	XP_041983199	539	*Aricia agestis*	Generalist	Lycaenidae	*Helianthemum nummulariu,* Geraniaceae	transmembrane protease serine 9-like	na
89	FJ205440	ACR16004	261	*Mamestra configurata*	Generalist	Noctuidae	*Canola sp.*	serine protease 2	Midgut
90	AY587154	AAT95346	219	*Sesamia nonagrioides*	Generalist	Noctuidae	*Zea mays*	trypsin Ia2, partial	Midgut
91	AY587155	AAT95347	257	*Sesamia nonagrioides*	Generalist	Noctuidae	*Zea mays*	trypsin Ia precursor	Midgut
92	AY587152	AAT95344	219	*Sesamia nonagrioides*	Generalist	Noctuidae	*Zea mays*	trypsin Ia2, partial	Midgut
93	XM_021338512	XP_021194195	755	*Helicoverpa armigera*	Generalist	Noctuidae	*Gossypium sp.*	transmembrane protease serine 9-like	Whole body
94	JH668299	KAG8110403	504	*Spodoptera frugiperda*	Generalist	Noctuidae	Poaceae, Asteraceae, Fabaceae	hypothetical protein SFRUCORN_012981	Whole body

95	JN793548	AFO68328	256	*Heliothis virescens*	Generalist	Noctuidae	*Nicotiana sp.*	Trypsin mRNA	Midgut
96	EF600059	ABU98624	260	*Helicoverpa armigera*	Generalist	Noctuidae	*Gossypium sp.*	Protease mRNA	Gut
97	AF045138	AAC02216	197	*Helicoverpa armigera*	Generalist	Noctuidae	*Gossypium sp.*	putative trypsin, partial	Midgut
98	AF261982	AAF74744	197	*Helicoverpa zea*	Generalist	Noctuidae	*Zea mays, Gossypium sp.*	Trypsin precursor mRNA	Midgut
99	AF261981	AAF74743	211	*Helicoverpa zea*	Generalist	Noctuidae	*Zea mays, Gossypium sp.*	Trypsin precursor mRNA	Midgut
100	KZ150010	PZC75087	260	*Helicoverpa armigera*	Generalist	Noctuidae	*Gossypium sp., Cajanus cajan, Cicer arietinum*	hypothetical protein B5X24_HaOG200380	na
101	**KY675288 (E8)**	**ASX32228**	**152**	***Samia ricini***	**Generalist**	**Saturniidae**	***Ricinus communis***	**Putative trypsin mRNA**	**gDNA**
102	XM_026889981	XP_026745782	827	*Trichoplusia ni*	Generalist	Noctuidae	Crucifers	uncharacterized protein LOC113507126	ovarian cell line Hi14
103	XM_035597205	XP_035448184	257	*Spodoptera frugiperda*	Generalist	Noctuidae	*Hordeum sp.*	trypsin, alkaline C-like	Whole body
104	KR024677	ALE15219	288	*Diatraea saccharalis*	Generalist	Crambidae	*Saccharum officinarum*	Chymotrypsin-like serine protease precursor	Midgut
105	JQ904142	AFM77772	313	*Ostrinia nubilalis*	Generalist	Crambidae	*Zea mays*	putative chymotrypsin 13	Unknown
106	KX380992	ANS56508	288	*Antheraea yamamai*	Generalist	Saturniidae	*Quercus sp.*	chymotrypsin-like serine protease 3	Whole organism
107	FJ217717	ACI45401	153	*Antheraea assama*	Generalist	Saturniidae	*Persea bombycina*	Putative chymotrypsin mRNA	Midgut
108	FJ217720	ACI45404	153	*Antheraea assama*	Generalist	Saturniidae	*Persea bombycina*	Putative chymotrypsin mRNA	Midgut
109	KX380990	ANS56506	290	*Antheraea yamamai*	Generalist	Saturniidae	*Quercus sp.*	chymotrypsin-like serine protease 1	Whole organism
110	KX380993	ANS56509	294	*Antheraea yamamai*	Generalist	Saturniidae	*Quercus sp.*	chymotrypsin-like serine protease 4	Whole organism

111	KX380994	ANS56510	271	*Antheraea yamamai*	Generalist	Saturniidae	*Quercus sp.*	chymotrypsin-like serine protease 5, partial	Whole organism
112	KF039687	AGU27161	284	*Antheraea pernyi*	Generalist	Saturniidae	*Quercus sp.*	Serine protease 13 mRNA	Hemolymph
113	FJ217716	ACI45400	157	*Antheraea assama*	Generalist	Saturniidae	*Litsea monopetala*	Putative chymotrypsin mRNA	Midgut
114	FJ217718	ACI45402	156	*Antheraea assama*	Generalist	Saturniidae	*Litsea monopetala*	Putative chymotrypsin mRNA	Midgut
115	FJ217719	ACI45403	158	*Antheraea assama*	Generalist	Saturniidae	*Litsea monopetala*	Putative chymotrypsin mRNA	Midgut
116	AB264092	BAF43531	440	*Samia ricini*	Generalist	Saturniidae	*Ricinus communis*	serine proteinase	na
117	AY829818	AAV91432	519	*Lonomia obliqua*	Generalist	Saturniidae	*Pyrus sp.*	Serine protease mRNA	Tegument
118	XM_021330937	XP_021186612	395	*Helicoverpa armigera*	Generalist	Noctuidae	*Gossypium sp.*	serine protease 3-like	Whole body
119	HM209426	ADI32887	254	*Helicoverpa armigera*	Generalist	Noctuidae	*Gossypium sp.*	Serine protease mRNA	Whole larvae
120	JX195651	AFQ37934	254	*Helicoverpa punctigera*	Generalist	Noctuidae	*Nicotiana sp.*	trypsinogen, partial	Gut
121	EU000257	ABW37094	199	*Heliothis virescens*	Generalist	Noctuidae	*Nicotiana sp.*	Putative trypsin-like mRNA	Midgut
122	EF531632	ABR88243	254	*Heliothis virescens*	Generalist	Noctuidae	*Nicotiana sp.*	Trypsin mRNA	Midgut
123	JN793542	AFO68322	255	*Heliothis virescens*	Generalist	Noctuidae	*Nicotiana sp.*	trypsin	Midgut
124	XM_042118937	XP_041974871	256	*Aricia agestis*	Generalist	Lycaenidae	*Helianthemum nummulariu,* Geraniaceae	trypsin CFT-1-like isoform X1	na
125	P35042	P35042	256	*Choristoneura fumiferana*	Specialist	Tortricidae	Pinaceae	Trypsin CFT-1	na
126	XM_028303299	XP_028159100	255	*Ostrinia furnacalis*	Generalist	Crambidae	*Zea mays*	trypsin CFT-1-like	Pupae

127	CAJQZP010000 693	CAG4977667	257	*Parnassius apollo*	Generalist	Papilionidae	*Papaveraceae, Crassulaceae*	unnamed protein product	na
128	AF064525	AAC36247	261	*Plodia interpunctella*	Generalist	Pyralidae	Cereals, legumes	Trypsin mRNA	Midgut
129	XM_026883284	XP_026739085	255	*Trichoplusia ni*	Generalist	Noctuidae	Crucifers	trypsin CFT-1-like	ovarian cell line Hi12
130	XM_037445818	XP_039752824	256	*Pararge aegeria*	Generalist	Nymphalidae	Poaceae	trypsin CFT-1-like	na
131	JACWZX01000 0176	KAG7310444	255	*Plutella xylostella*	Specialist	Plutellidae	Brassicaceae	hypothetical protein JYU34_003225	Pupa
132	XM_013277673	XP_013133127	256	*Papilio polytes*	Specialist	Papilionidae	*Citrus sp.*	trypsin, alkaline C-like	na
133	KQ460226	KPJ16474	501	*Papilio machaon*	Specialist	Papilionidae	*Citrus sp.*	Trypsin CFT-1	Whole body
134	XM_014501040	XP_014356526	256	*Papilio machaon*	Specialist	Papilionidae	*Pimpinella saxifrage*	trypsin, alkaline C-like	Whole body
135	AF173498	AAF24228	260	*Plodia interpunctella*	Generalist	Pyralidae	Cereals, legumes	Trypsin-like precursor	Midgut
136	AF173497	AAF24227	260	*Plodia interpunctella*	Generalist	Pyralidae	Cereals, legumes	Trypsin-like precursor	Midgut
137	XM_013330814	XP_013186268	262	*Amyelois transitella*	Generalist	Pyralidae	Fig, walnut, almond, pistachio	trypsin, alkaline C-like isoform X1	na
138	XM_013310431	XP_013165885	260	*Papilio xuthus*	Specialist	Papilionidae	*Citrus sp.*	trypsin, alkaline C-like	na
139	AM690450	CAM84320	273	*Manduca sexta*	Specialist	Sphingidae	Solanaceae	Trypsinogen-like mRNA	Midgut
140	XM_026889982	XP_026756600	261	*Galleria mellonella*	Generalist	Pyralidae	*Figus sp.*	trypsin, alkaline C-like	whole adult
141	XM_034980299	XP_034836190	262	*Maniola hyperantus*	Generalist	Nymphalidae	Poaceae	trypsin, alkaline C-like	Multiple Hosts
142	XM_022959264	XP_023934507	261	*Bicyclus anynana*	Generalist	Nymphalidae	Poaceae	trypsin, alkaline C-like	Whole body
143	XM_026627408	XP_026483193	224	*Vanessa tameamea*	Specialist	Nymphalidae	*Urticceae*	trypsin, alkaline C-like	Thorax

144	AY513649	AAR98919	257	*Ostrinia nubilalis*	Specialist	Crambidae	*Zea mays*	Trypsin-like proteinase	Unknown
145	JTDY01000728	KOB76086	854	*Operophtera brumata*	Generalist	Geometridae	*Quercus sp.*	Trypsin	head and thorax
146	RSAL01000066	RVE49371	328	*Chilo suppressalis*	Specialist	Crambidae	*Oryza sativa*	hypothetical protein evm_005986	whole body
147	JX866720	AFW03963	257	*Diatraea saccharalis*	Generalist	Crambidae	*Saccharum officinarum*	trypsin 2	na
148	XM_028303288	XP_028159089	258	*Ostrinia furnacalis*	Generalist	Crambidae	*Zea mays*	trypsin, alkaline C-like	Pupae
149	XM_026463618	XP_026319403	515	*Hyposmocoma kahamanoa*	Generalist	Cosmopterigidae	Algae or lichen	transmembrane protease serine 9-like	Whole body
150	XM_026464478	XP_026320263	265	*Hyposmocoma kahamanoa*	Generalist	Cosmopterigidae	*Algae or lichen*	trypsin, alkaline A-like	Whole body
151	HM011050	ADG26770	261	*Antheraea pernyi*	Generalist	Saturniidae	*Quercus sp.*	cocoonase-like protein	na
152	AB264091	BAF43530	438	*Samia ricini*	Generalist	Saturniidae	*Ricinus communis*	prophenoloxidase-activating proteinase	na
153	AB678221	BAL04888	274	*Antheraea yamamai*	Generalist	Saturniidae	*Quercus sp.*	Serine protease like mRNA	Reproductive organ
154	AB678222	BAL04889	274	*Samia cynthia pryeri*	Generalist	Saturniidae	*Paulownia sp.*	Serine protease like mRNA	Reproductive organ
155	AB678223	BAL04890	274	*Samia cynthia ricini*	Generalist	Saturniidae	*Ricinus communis*	Serine protease like mRNA	Reproductive organ
156	AB684323	BAM15953	275	*Rhodinia fugax*	Generalist	Saturniidae	*Quercus sp.*	Serine protease like mRNA	Reproductive organ
157	AB738397	BAM34530	274	*Actias artemis*	Generalist	Saturniidae	*Salix, Alnus*	Serine protease likemRNA	Reproductive organ
158	AB684324	BAM15954	274	*Saturnia jonasii*	Generalist	Saturniidae	*Prunus sp.*	Serine protease like mRNA	Reproductive organ
159	AB738398	BAM34531	274	*Antheraea pernyi*	Generalist	Saturniidae	*Quercus sp.*	Serine protease like mRNA	Reproductive organ
160	AB924381	BAP76070	273	*Aglia japonica*	Generalist	Saturniidae	*Quercus, Betula*	Serine protease like protein	Reproductive organ

161	DQ872511	ABI50224	159	*Antheraea assamensis*	Generalist	Saturniidae	Lauraceae	Putative trypsin	Midgut
162	FJ217713	ACI45397	159	*Antheraea assama*	Generalist	Saturniidae	*Persea bombycina*	Putative trypsin mRNA	Midgut
163	AY829841	AAV91455	280	*Lonomia obliqua*	Generalist	Saturniidae	*Pyrus sp.*	Serine protease mRNA	Spicule
164	AY829842	AAV91456	216	*Lonomia obliqua*	Generalist	Saturniidae	*Pyrus sp.*	Serine protease mRNA	Spicule
165	AY829843	AAV91457	315	*Lonomia obliqua*	Generalist	Saturniidae	*Pyrus sp.*	Serine protease mRNA	Spicule
166	XM_004925968	XP_004926025	553	*Bombyx mori*	Specialist	Bombycidae	*Morus alba*	digestive cysteine proteinase 1 isoform X2	na
na- information not available									
