# Supplementary material for: Midgut serine proteinases participate in dietary adaptations of the castor (Eri) silkworm Samia ricini Anderson transferred from Ricinus communis to an ancestral host, Ailanthus excelsa Roxb
Source: Front Insect Sci. 2023 Aug 10;3:1169596. doi: 10.3389/finsc.2023.1169596 (PMC10926435; doi:10.3389/finsc.2023.1169596)
Supplement: Supplementary file 1 [file DataSheet_1.zip › 1. Revised Figure legends Supplementary information.docx]

**Supplementary Figures**

Figure S1.1. Estimation of nutritional indices and relative growth rate (RGR) of *S. ricini* from neonate to fifth instar larvae reared continuously on *R. communis* (Scr) and *A. excelsa* (Scai) diets*.* Abbreviations are the same as in Figure 2. Bars depict mean ± SE. Significant differences at p≤0.05 are denoted by different alphabets.

Figure S1.2. Images of third instar larvae of *S. ricini* feeding on (A) *R. communis* and (B) *A. excelsa*; (C) cocoons from insects reared on (left two) *R. communis* and (right two) *A. excelsa*;

(D) a female moth and (E) a male moth.

Figure S1.3. Box plots show range of weights of fifth instar larva and pupa of *S. ricini* reared on *R. communis* (Scr) and *A. excelsa* (Scai). Horizontal lines within each box represents the median larval and pupal weights (50^th^ percentile) while the whiskers represent minimum and maximum values for each trait.

Figure S1.4. Eri sericulture as practiced in family-run enterprises. Images show (A) larvae of *S. ricini* (Eri) feeding on *R. communis*; (B) Eri cocoons on a long wooden tray; (C) reeling machines used for Eri silks; (D) threads of Eri cocoon silk in bundles; (E) typical wooden loom used to weave Eri silk yarn.

Figure S2. Gelatinolytic zymograms of midgut proteases from fourth instar *S. ricini* fed on *R. communis* (Scr) diet upon incubation with protease inhibitors STI, SBBI, leupeptin, Aprotinin, E-64, EDTA, and pepstatin. Lanes BT refer to bovine trypsin. Details of concentrations used are provided in Materials and methods. Activity zones of different mobility are denoted with a star.

Figure S3A. Semi-degenerate PCR primer-pairs used for amplification of lepidopteran serine proteinase genes based on conserved regions flanking the catalytic H57 and S195 (numbering after Bovine chymotrypsin). The forward primer (DmTF) was built around residues encompassing H57 in the α-trypsin gene of *Drosophila melanogaster* (Accession# P04814). Reverse PCR primers (DmTR/RcTR) were built around K/R188 residues that precede the D189 residue within the specificity pocket of trypsins (45). A generic reverse primer (SerPr) encoding the S195 residue after Mazumdar-Leighton et al (44) is also shown. Since the majority of amino acids in mammalian serine proteinases (and models of lepidopteran enzymes) that interact with plant PIs occur downstream of the conserved H57 residue, and are concentrated around positions 188 and 190 of serine proteases (14, 45, Dias *et al*., 2015), these primer pairs provide a useful foray into sequence diversity of serine protease gene family in phytophagous Lepidoptera. Relative occurrence of amino acids in serine proteases identified in *S. ricini* using these primers were visualized using Jalview ver 2.11.2.2 ([www.jalview.org/](http://www.jalview.org/)). The residues are coloured according to the default scheme used for Clustal Omega ([www.ebi.ac.uk/Tools/msa/clustalo/](http://www.ebi.ac.uk/Tools/msa/clustalo/)).

Figure S3B. Restriction fragment length polymorphisms in RNA-PCR amplicons encoding putative serine proteinases in midguts of fourth instar *S. ricini* feeding on Scr diet (Rc) and Scai diet (Ai). A 3% agarose gel stained with ethidium bromide (0.05µg/ml) shows results obtained with the 4-base cutters *Alu* I and *Hinf* I. Primer pairs used were: (P1) DmTF/SerPR; (P2) DmTF/DmTR; and (P3) DmTF/RcTR. Lanes M and M100 denote 1kb DNA ladder (Fermentas, USA, catalog# SM0312) and 100bp DNA ladder (Fermentas, USA, catalog#SM0241) respectively. The arrowhead denotes DNA fragments of 500bp.

Figure S4. Alignment of putative serine proteases encoded by cDNA fragments from midguts of fourth instar *S. ricini* using MEGA 7.0 ([www.megasoftware.net](http://www.megasoftware.net)). Conserved motifs around active site residues H57, D102 and S195 (overhead arrows) are shown for groups/lineages Sr I -Sr VIII. Closely-related lepidopteran orthologs from BLAST-p search results and best-match invertebrate/mammalian homologs from structural database, SWISSPROT (retrieved on 23^rd^ June 2022) are also displayed. Color codes for amino acids are as provided in Clustal Omega ([www.ebi.ac.uk/Tools/msa/clustalo/](http://www.ebi.ac.uk/Tools/msa/clustalo/)).
