# Supplementary figures and images for: Midgut serine proteinases participate in dietary adaptations of the castor (Eri) silkworm Samia ricini Anderson transferred from Ricinus communis to an ancestral host, Ailanthus excelsa Roxb
Source: Front Insect Sci. 2023 Aug 10;3:1169596. doi: 10.3389/finsc.2023.1169596 (PMC10926435; doi:10.3389/finsc.2023.1169596)

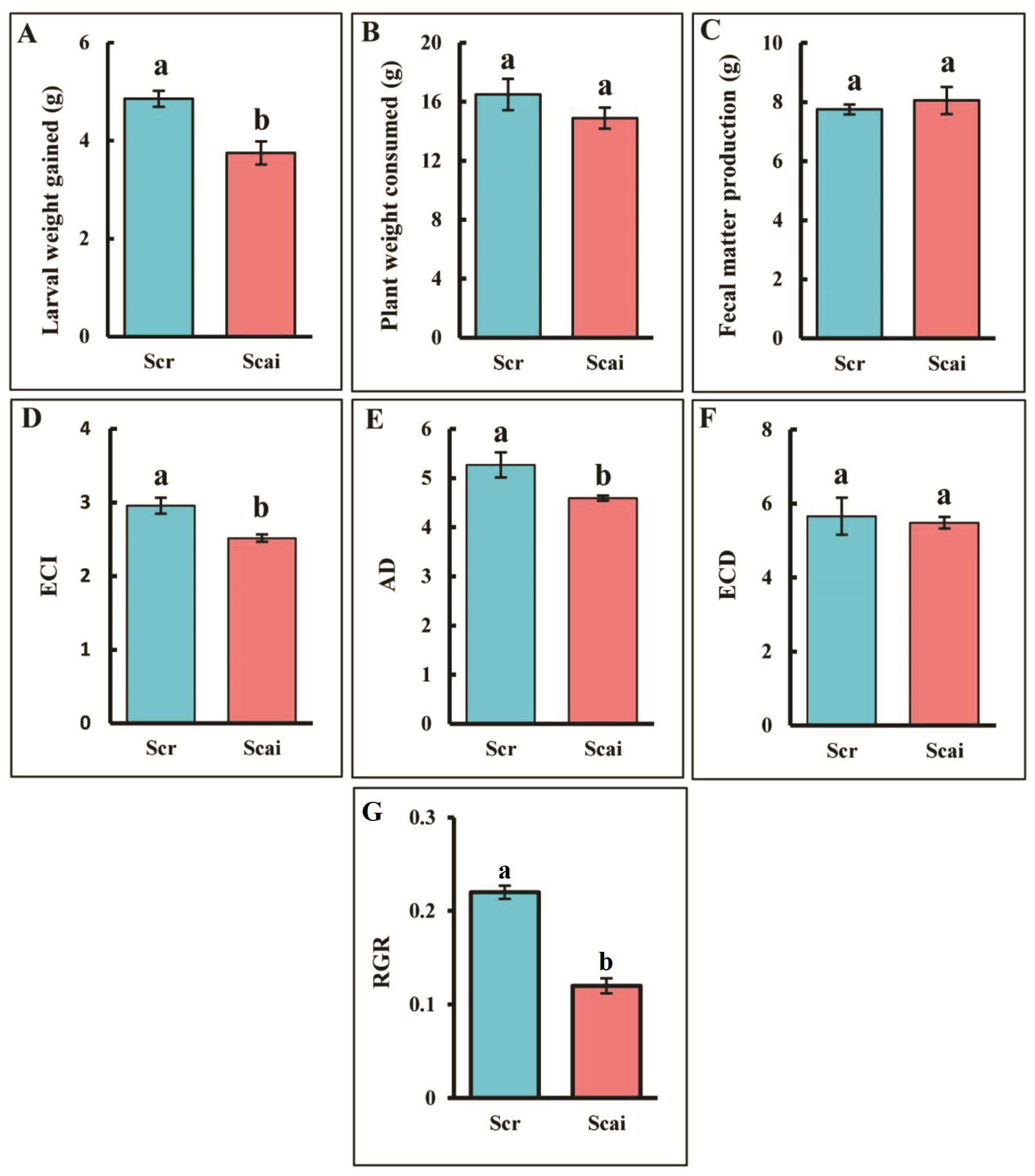

Supplement: Supplementary file 1 [file DataSheet_1.zip › Figure S1.1.tif]

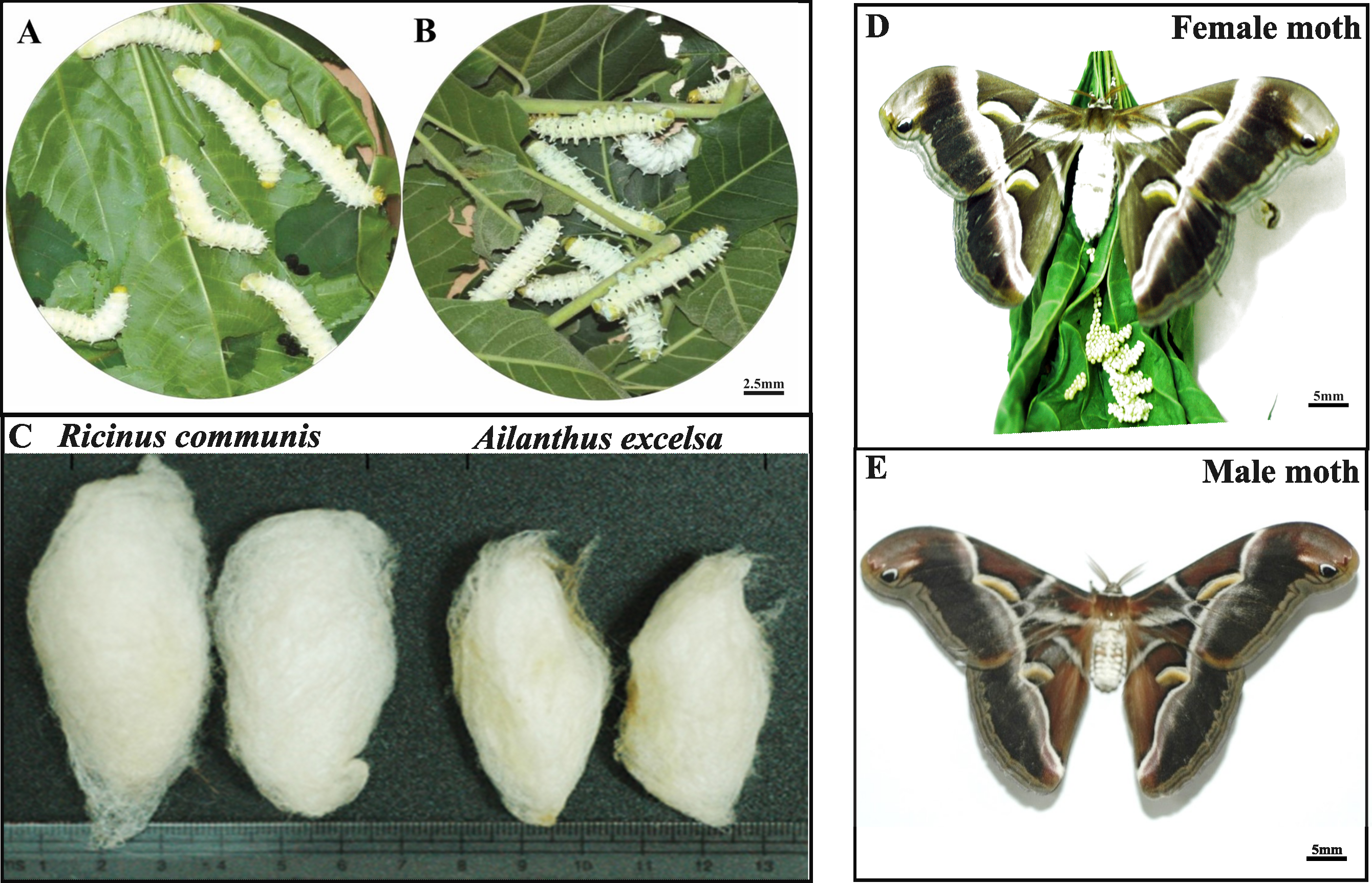

Supplement: Supplementary file 1 [file DataSheet_1.zip › Figure S1.2.tiff]

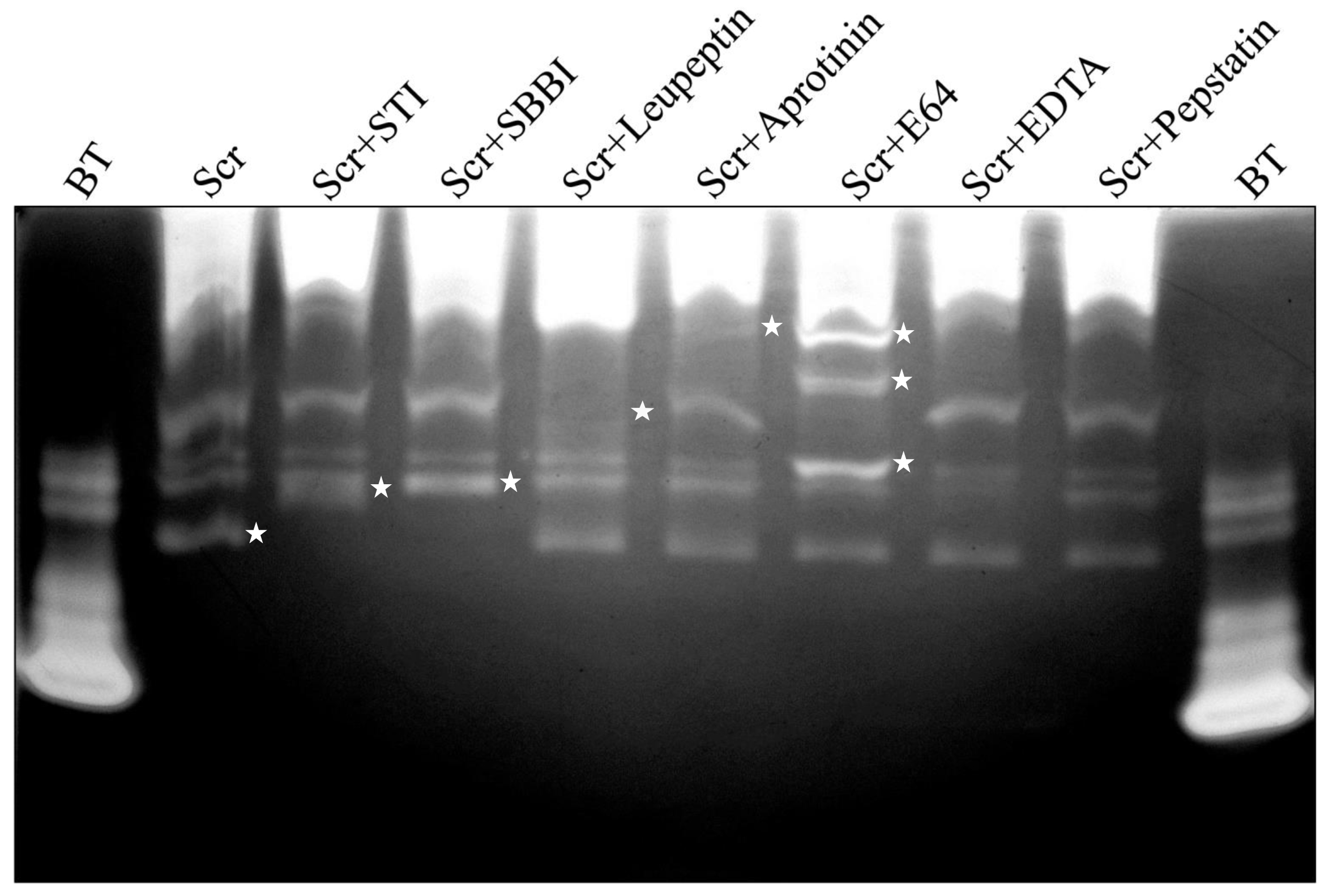

Supplement: Supplementary file 1 [file DataSheet_1.zip › Figure S2.tiff]

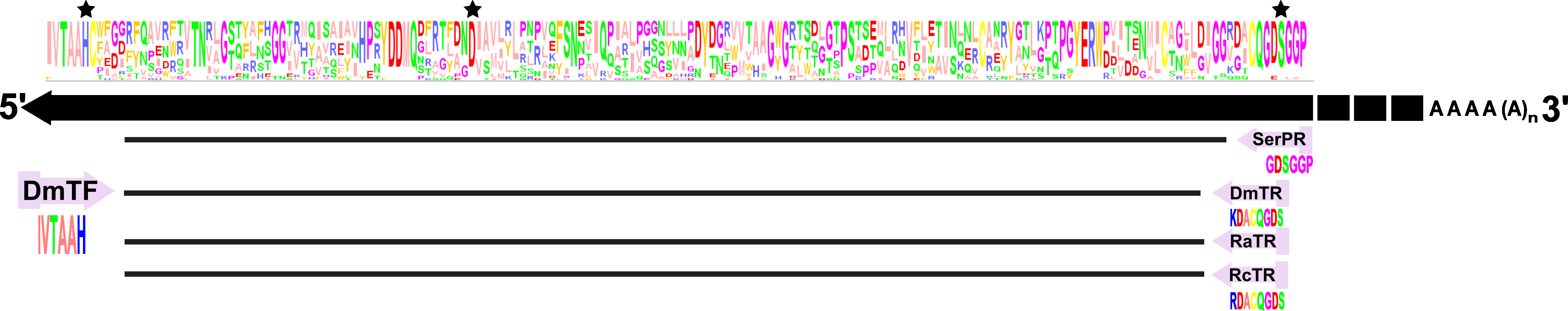

Supplement: Supplementary file 1 [file DataSheet_1.zip › Figure S3A.tiff]

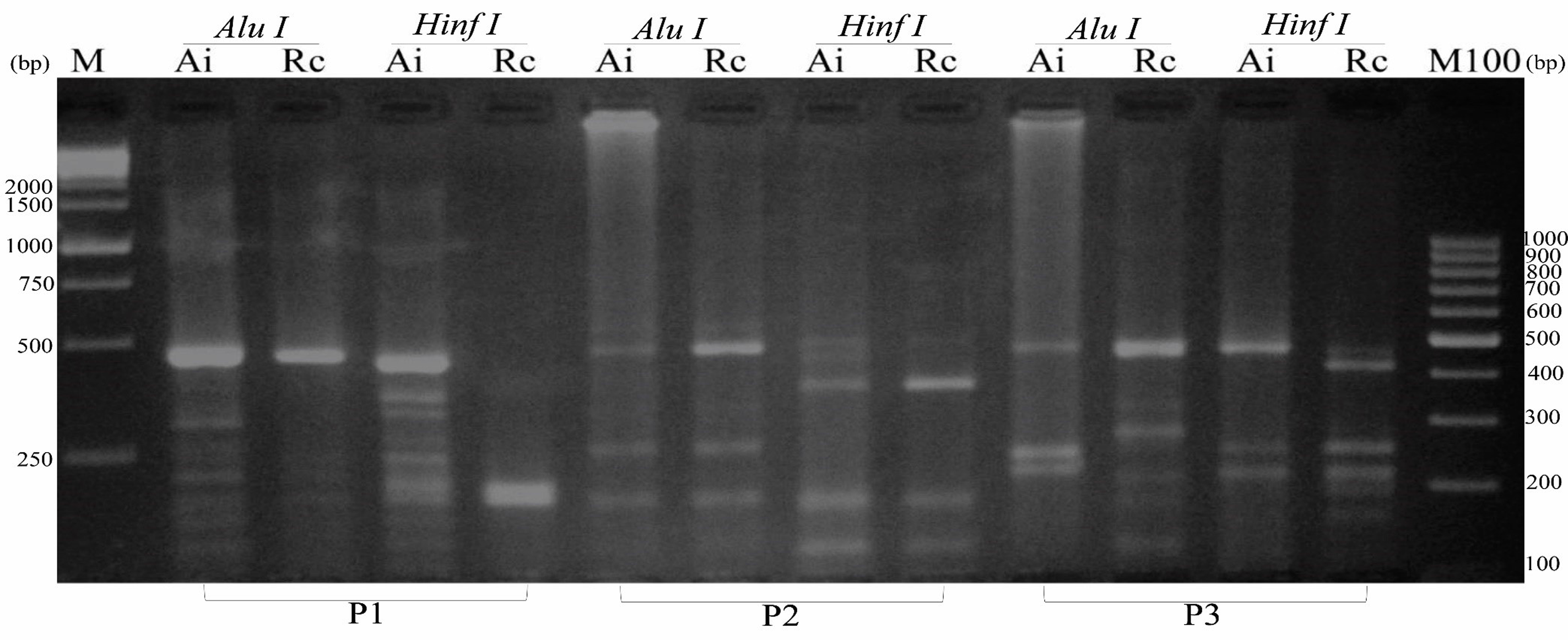

Supplement: Supplementary file 1 [file DataSheet_1.zip › Figure S3B.tif]

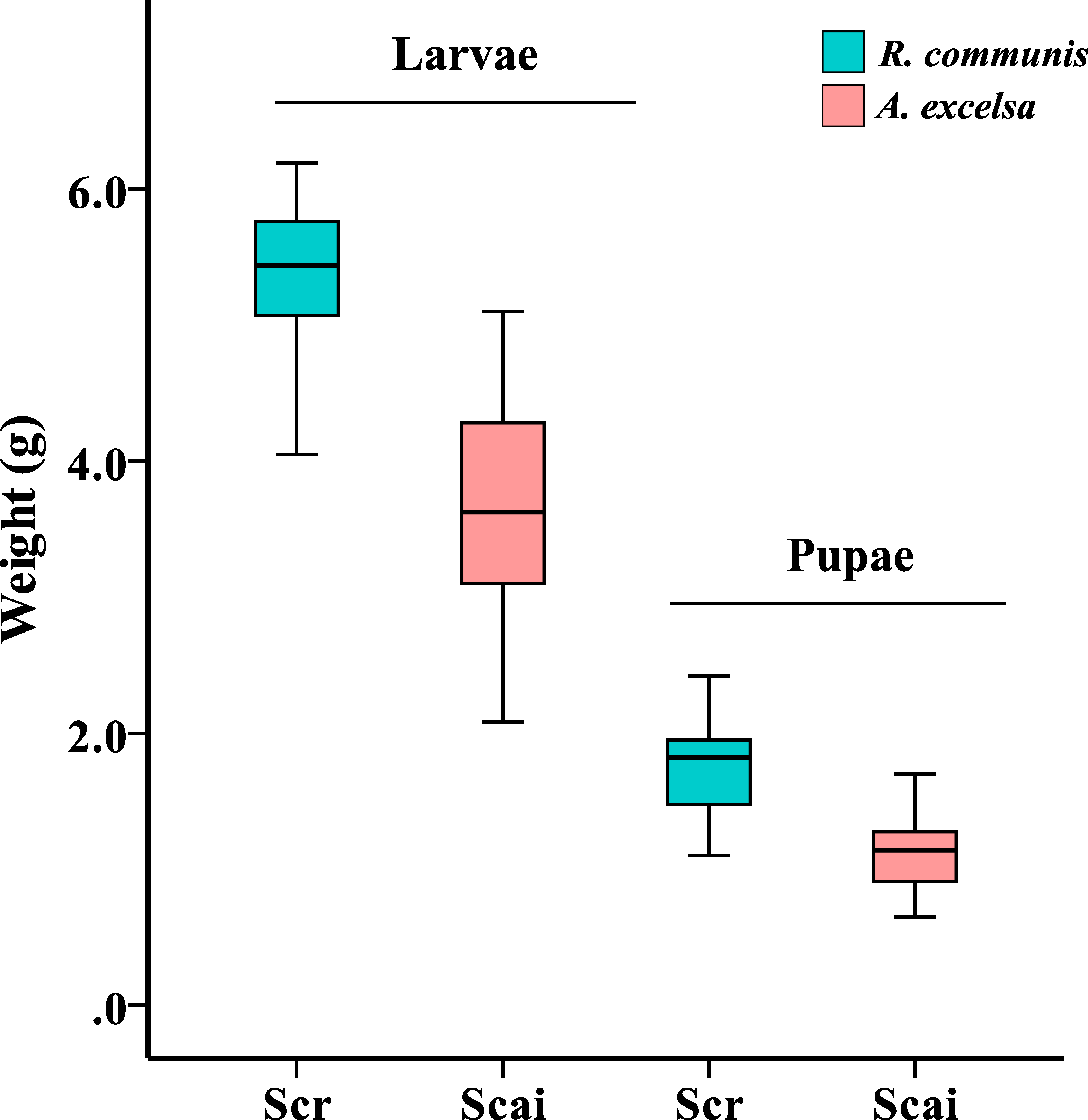

Supplement: Supplementary file 1 [file DataSheet_1.zip › Figure S1.3.tiff]

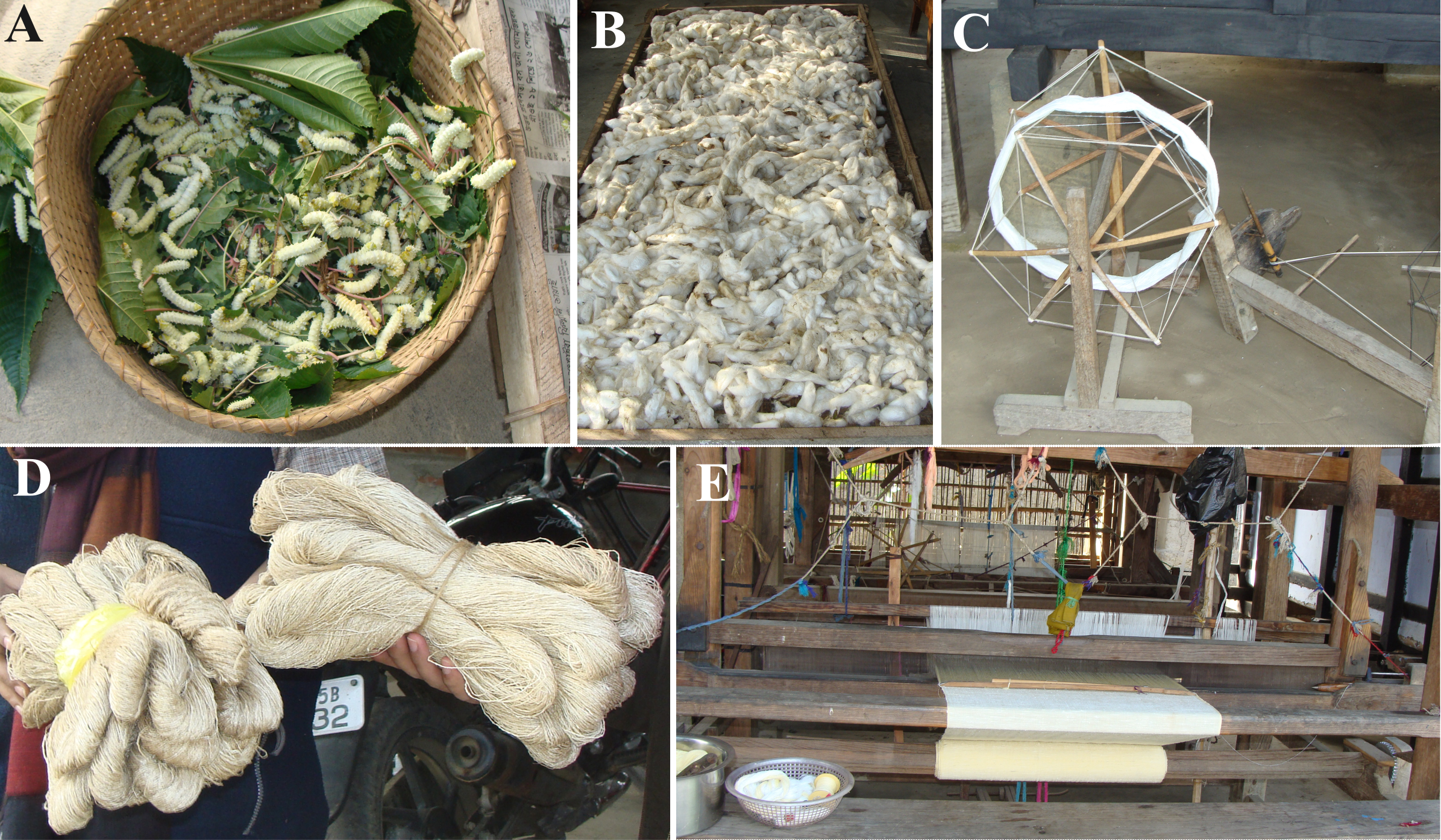

Supplement: Supplementary file 1 [file DataSheet_1.zip › Figure S1.4.tiff]

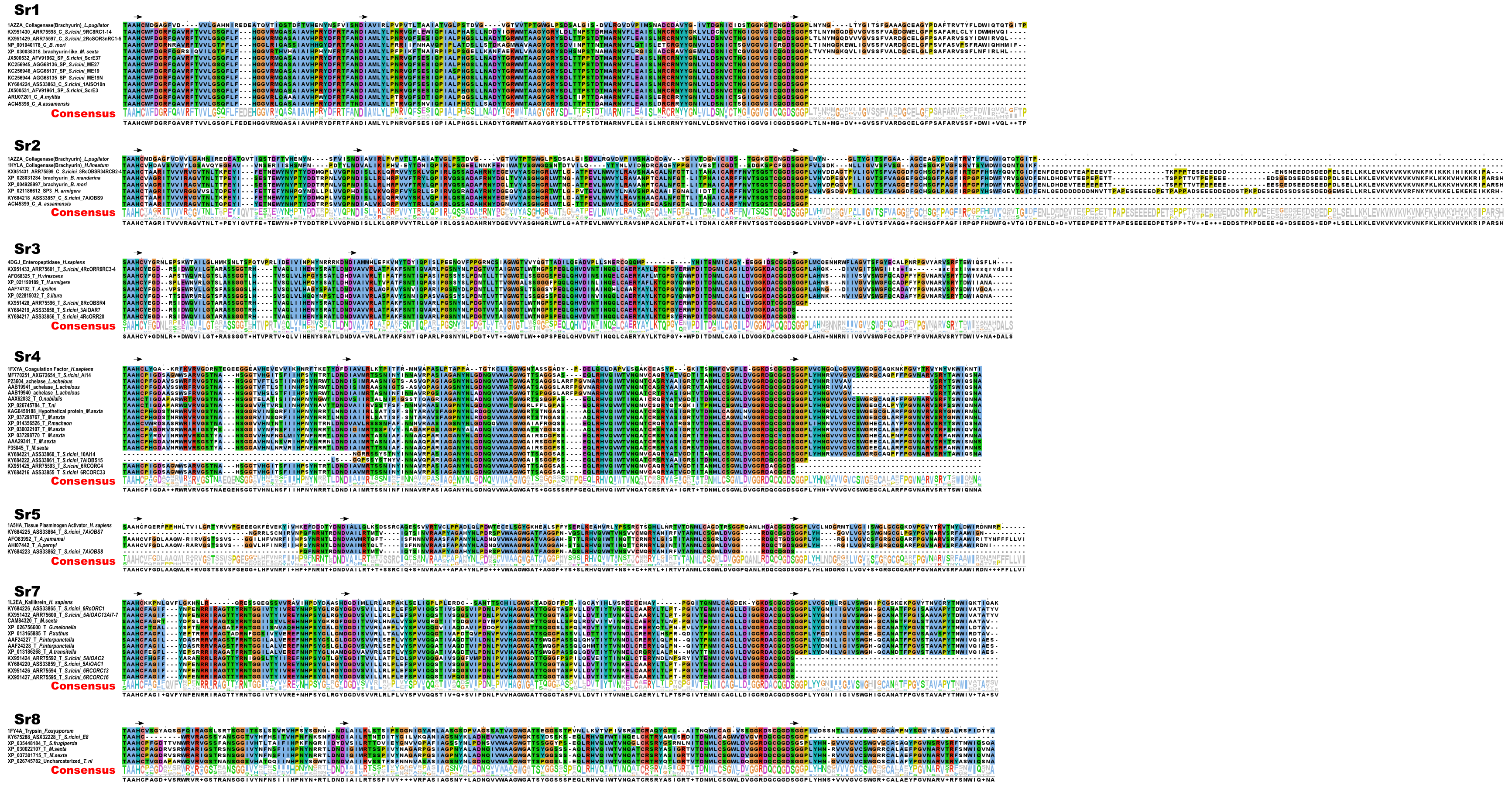

Supplement: Supplementary file 1 [file DataSheet_1.zip › Figure S4.tif]
